# Visualizing Late Insect Embryogenesis: Extraembryonic and Mesodermal Enhancer Trap Expression in the Beetle *Tribolium castaneum*

**DOI:** 10.1371/journal.pone.0103967

**Published:** 2014-07-31

**Authors:** Stefan Koelzer, Yvonne Kölsch, Kristen A. Panfilio

**Affiliations:** Institute for Developmental Biology, University of Cologne, Cologne, Germany; University of Oxford, United Kingdom

## Abstract

The beetle *Tribolium castaneum* has increasingly become a powerful model for comparative research on insect development. One recent resource is a collection of *piggyBac* transposon-based enhancer trap lines. Here, we provide a detailed analysis of three selected lines and demonstrate their value for investigations in the second half of embryogenesis, which has thus far lagged behind research on early stages. Two lines, G12424 and KT650, show enhanced green fluorescent protein (EGFP) expression throughout the extraembryonic serosal tissue and in a few discrete embryonic domains. Intriguingly, both lines show for the first time a degree of regionalization within the mature serosa. However, their expression profiles illuminate distinct aspects of serosal biology: G12424 tracks the tissue’s rapid maturation while KT650 expression likely reflects ongoing physiological processes. The third line, G04609, is stably expressed in mesodermal domains, including segmental muscles and the heart. Genomic mapping followed by *in situ* hybridization for genes near to the G04609 insertion site suggests that the transposon has trapped enhancer information for the *Tribolium* orthologue of *midline* (*Tc-mid*). Altogether, our analyses provide the first live imaging, long-term characterizations of enhancer traps from this collection. We show that EGFP expression is readily detected, including in heterozygote crosses that permit the simultaneous visualization of multiple tissue types. The tissue specificity provides live, endogenous marker gene expression at key developmental stages that are inaccessible for whole mount staining. Furthermore, the nonlocalized EGFP in these lines illuminates both the nucleus and cytoplasm, providing cellular resolution for morphogenesis research on processes such as dorsal closure and heart formation. In future work, identification of regulatory regions driving these enhancer traps will deepen our understanding of late developmental control, including in the extraembryonic domain, which is a hallmark of insect development but which is not yet well understood.

## Introduction

After the fruit fly *Drosophila melanogaster*, the red flour beetle, *Tribolium castaneum*, is one of the most established models for studying insect development. In recent years, many advances have been made in building the toolkit for *Tribolium* research, including genomic resources, forward and reverse genetics techniques, and transgenesis for both visualization and mis-expression applications [1]–[6]. These tools make *Tribolium* a powerful resource for comparative developmental genetics, as the biology of this species is less derived and thus more broadly representative of insect development than *Drosophila*. For example, insights have been obtained regarding features that are not present in *Drosophila*, including short germ axial patterning and the development of embryonic appendages and an un-everted head [7]–[12].

However, a majority of these studies have focused on early development or specific anatomical structures, while much of later embryogenesis remains to be examined. One striking way in which later development differs between *Drosophila* and *Tribolium* is in the extraembryonic tissue complement. While *Drosophila* eggs have an amnioserosa, a monolayered epithelium that covers the yolk dorsally, most insects, including *Tribolium*, have distinct amniotic and serosal tissues that cover both the embryo and yolk [13], [14]. Their late morphogenetic movements to withdraw from the embryo are both essential for development in *Tribolium* and more representative of how these tissues play key roles in positioning the embryo itself, also in the more basally branching hemimetabolous insects [13], [15], [16].

Furthermore, there are two key challenges to the visualization of late development in *Tribolium*. Firstly, many known genes with early tissue specification roles are not persistent tissue markers, either because they are not expressed at later stages or because expression is spatially dynamic (*e.g.*, *pannier* [10]). Secondly, the extraembryonic serosa secretes a robust cuticle that adheres to the vitelline membrane of the eggshell [17], [18]. This cuticle forms relatively early, effectively blocking staining reagents for visualization in whole mount, undamaged eggs throughout most of embryogenesis (from approximately 22% to 72% development, encompassing late germband extension until serosal rupture/dorsal organ stages). Shortly after this, the prolarval cuticle of the embryo similarly obstructs staining. Thus, there is a need for new means of examining late developmental processes.

Here, we build on one of the recent innovations in *Tribolium* to address this need. A transposon insertion screen generated a number of enhancer trap lines through the random insertion of an enhanced green fluorescent protein (EGFP) expressing construct into the genome [5]. The outcome of this “GEKU” screen, named after the initials of the participating laboratories, is publicly available through a dedicated web site (see Methods).

For this study, we selected three enhancer trap lines with potential extraembryonic or late dorsal embryonic expression for detailed analysis – two with serosal expression and one that labels the heart and a subset of mesodermal and sensory structures – and also mapped the insertion sites. As we show, the serosal lines strongly label this tissue throughout its development, including at those stages inaccessible for whole mount staining, and they provide the first evidence for spatio-temporal regionalization within the tissue. At the same time, the differing expression profiles of the two lines correlate with different aspects of serosa development. In the case of the mesodermal line, EGFP labels a series of landmark embryonic positions along both the anterior-posterior and dorsal-ventral axes. These features make the selected lines strong tools for visualizing and analyzing late embryogenesis in *Tribolium*, particularly in applications for our ongoing examination of late extraembryonic morphogenesis, including the process of dorsal closure [16]. Furthermore, our examinations highlight key ways in which this enhancer trap collection provides a valuable resource for future developmental research.

## Results

As described in the original screening paper [5], the enhancer trap lines are homozygous for the pBac{3xP3-EGFPaf} transposon cassette, which contains the coding sequence for EGFP under the artificial 3xP3 promoter with three binding sites for Pax6 [19], [20]. Hence, all lines have fluorescence in the eyes as a marker for transgenesis, in addition to new expression domains resulting from enhancer trapping. Below, we present the EGFP expression patterns of the three selected lines and evaluate the genomic contexts into which the transposon inserted.

### Developmental staging calibration for live imaging across embryogenesis

**Figure 1:**
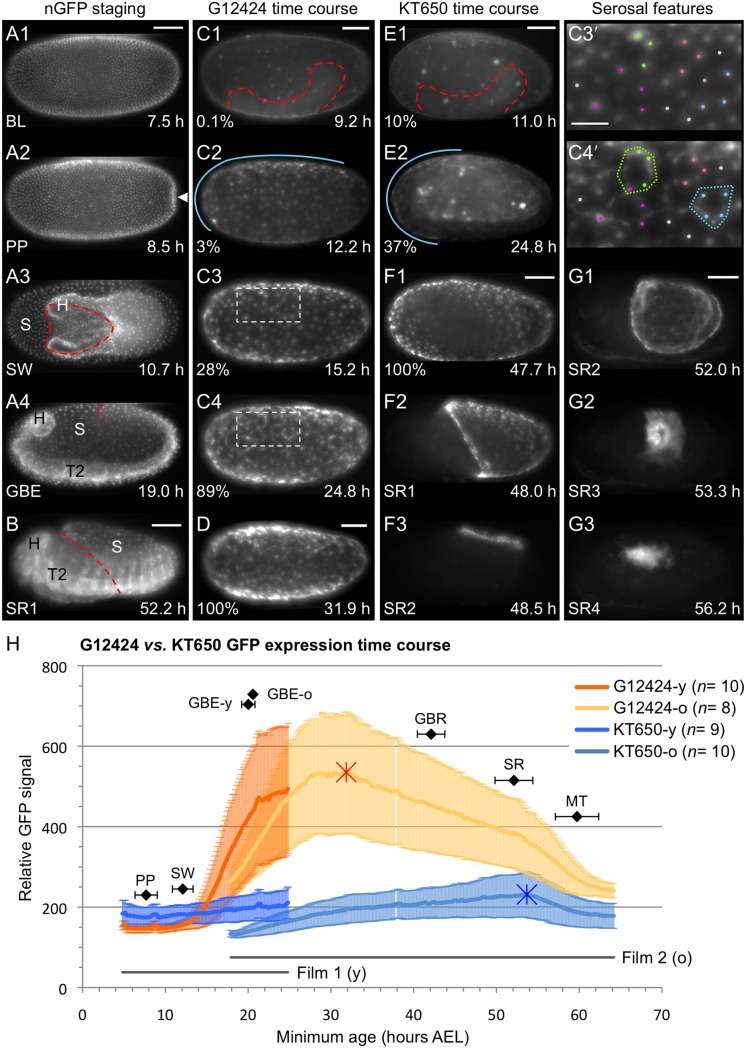
Progression of serosal expression in the lines G12424 and KT650 across embryogenesis. Live imaging detection of GFP in the transgenic *Tribolium* lines nGFP, G12424, and KT650. Micrographs labeled with the same letter are of a single embryo. Views are ventral (A1**–**A3), lateral (A4,B,C,E,F), or dorsal (D,G), with anterior left. (**A–B**) Exemplar nGFP embryos, illustrating the uniform blastoderm (BL), primitive pit (PP, white arrowhead), closing serosal window (SW), maximum germband extension (GBE), and mid serosal rupture (SR1) stages. Dashed lines label the serosal edge (A3,B) and posterior abdomen (A4). The embryo in panels A1–4 naturally rotated to a lateral view. (**C–D**) G12424 serosal EGFP increases from shortly after serosal window closure through mid germband retraction. (**E**–**F1**) KT650 serosal EGFP increases from shortly after maximum germband extension until just before serosal rupture. Both lines exhibit early EGFP expression in yolk globules (C1,E1: dashed outlines show embryo position, SW stage). The onset of serosal EGFP expression shows an anterior-dorsal or anterior bias (C2,E2: blue lines). During germband retraction, expression in both lines becomes dynamic, with streaks of EGFP between serosal nuclei (shown for G12424: compare C3′ and C4′, dots mark selected nuclei). (**F2**–**G3**) In both lines, serosal expression persists throughout the lifetime of the tissue (shown for KT650: sequential stages following serosal rupture, SR1-4, through tissue degeneration). Percentage values (C–F1) denote normalized EGFP intensity for each line. Time stamps show embryo minimum age (at 30°C). Anatomical abbreviations: H, head; S, serosa; T(x), thoracic segment (x). Scale bars are 100 µm, except for 50 µm in C3′. (**H**) Quantification of EGFP expression time courses, showing the mean ± standard deviation, with sample sizes indicated in the legend. Asterisks mark the maximum EGFP signal. Also plotted are the durations of the two films (younger, “y”; older, “o”: grey lines) and the morphological stages defined in Table 3 (black plot points). See Methods for details.

We took a live imaging approach to characterizing the enhancer trap EGFP expression. To contextualize the enhancer trap lines’ spatially and temporally restricted expression, in parallel we filmed embryos from a line that ubiquitously expresses nuclear-localized GFP (nGFP, [4]). This allowed us to precisely determine the timing of landmark morphological stages (Fig. 1A–B, Table 1). Briefly, gross morphogenesis and development of the external structures in the *Tribolium* egg proceed as follows. The cellularized epithelium of the **blastoderm** on the yolk surface differentiates into the serosa and the germ rudiment (embryo and amnion). The first morphological change is a small depression at the posterior pole, the **primitive pit**, which rapidly enlarges into a posterior fold. Fold outgrowth culminates in the internalization of the embryo relative to both extraembryonic membranes, where the constricting region through which the embryo is visible is termed the **serosal window** [3], [14], [21]. The embryo then proceeds to add body segments during **germband extension**, and then to thicken during **germband retraction** [17]. Subsequently, the **serosa ruptures** in the ventral-anterior region where it had previously completed closure, leading to its withdrawal and final degeneration during dorsal closure of the embryonic epidermis; during this process the folded, compacted serosa is known as the ‘dorsal organ’ [15]–[17]. After dorsal closure, continued maturation of the embryo includes increasing physical activity as the longitudinal body **muscles twitch** periodically (Table 1).

**Table 1:** Morphological staging definitions and values from quantitative time-lapse analyses at 30°C (see also Fig. 1).

Label	Stage name	Age^a^	*n*	Definition
BL	Blastoderm	Underway at filming start (4.8 h; 6.7%)^b^	38	Generation of a monolayered epithelium over the complete egg surface
PP	Primitive pit	7.7±1.3 (10.7±1.8%)	37	First detectable flattening of cells at the posterior pole
SW	Serosal window	12.1±1.3 (16.8±1.8%)	33	Closure of the serosal window over the embryo: detachment of serosa from internal tissue allows the abrupt anteriorward extension of the head
GBE	Extended germband	20.2±0.8 (28.1±1.1%)^c^	13	Maximum germband extension first attained
GBR	Retracted germband	42.1±1.7 (58.5±2.4%)	12	Complete germband retraction first attained
SR	Serosal rupture	52.1±2.3 (72.4±3.2%)	32	First detection of rupture at the onset of serosal withdrawal^d^
MT	Muscle twitches	59.7±2.6 (82.9±3.6%)	28	First detection of embryonic twitching due to lateral muscle activity

a Age is given in hours after egg lay for the minimum age from a 4-hour range, as the mean ± standard deviation. Parenthetical values for percent of development are based on a total embryogenesis period of 72 hours.

b As the complete blastoderm stage was not quantified, we do not provide a definitive staging landmark and hence do not plot this stage in Figure 1H.

c For GBE, data were pooled from movie recordings 1 and 2. The separate values are shown in Fig. 1H.

d Note that the staging landmark definition corresponds to the “SR” plot point in Figure 1H, whereas the designations SR1–SR4 in Figure 1 micrographs subdivide the subsequent morphological progression of serosal tissue contraction and withdrawal from the embryo.

### The lines G12424 and KT650 are expressed in the mature extraembryonic serosa

Live imaging examination of EGFP expression in the lines G12424 and KT650 was conducted simultaneously on samples sizes of 8–10 embryos per line per movie, for the period from mid blastoderm stage until after dorsal closure (Fig. 1; 4.8–64.2 hours after egg lay [hAEL], or 7–89% development: see Methods for details). Both lines exhibit early EGFP within the yolk before acquiring expression specifically within the serosa, although they differ in the intensity and onset of serosal expression.

From the earliest stages examined, both lines show low-level EGFP expression in dynamic yolk globules (Fig. 1C1,E1). Any one globule is only visible briefly within the cortical region before appearing to sink back into the yolk. We observed no spatial bias in the location of yolk EGFP but note that the size of the globules is variable and occasionally larger in the KT650 line. Yolk EGFP is also detectable for longer in this line, but this may simply reflect the relative loss of yolk signal compared to strengthening serosal signal, which occurs later in KT650. However, in both lines EGFP expression in the yolk ceases in later development: once the serosa contracts and exposes the yolk for direct visualization, no EGFP is detected in this domain (Fig. 1F2–F3,G1–G3).

The onset of serosal expression shows a slight but consistent asymmetry, with EGFP first detectable in a broad anterior-dorsal domain in G12424 and in an anterior cap domain in KT650 (Fig. 1C2,E2), before becoming expressed throughout the tissue (Fig. 1C3,C4,D,F1). Quantification of the EGFP signal (see Methods) reveals very different time courses to the expression profiles of the two lines (Fig. 1H). G12424 serosal EGFP is first detectable above background levels shortly after the serosal window closes and the serosa is a complete and intact tissue over the egg surface. EGFP intensity then increases rapidly during germband extension, culminating in peak expression at 31.9 hAEL (Fig. 1H: red asterisk), which is roughly the midpoint between germband extension and retraction (Table 1). From this peak of expression, serosal EGFP intensity wanes through the rest of embryogenesis, where EGFP in late (post-serosa) embryos is largely due to expression under the control of the 3XP3 promoter (see below). In contrast, KT650 serosal EGFP develops gradually (Fig. 1H). In our survey of early eggs, only seven of nine KT650 eggs exhibited clear serosal expression by the end of this period (Film 1: until shortly after the extended germband stage), and only one of these had advanced from the anterior pole onset to expression throughout the serosa. Discounting a slight increase in EGFP intensity as the ruptured serosa begins to consolidate (53.7 hAEL; Fig. 1H: blue asterisk), peak expression occurs just before rupture (0.2 h before). As the serosa then compacts and degenerates, EGFP intensity declines (Fig. 1F2–F3,G1–G3).

As suggested by the error bars in Fig. 1H (one standard deviation either side of the mean), there is quite a high degree of inter-embryo variability in EGFP intensity in both lines, although some of this reflects the four-hour (6% development) age range of the samples. The maximum EGFP signal differed between the strongest and weakest individual specimens by 2.3-fold in G12424 and by 1.8-fold in KT650. Despite this variability, the G12424 line clearly gives an earlier and stronger signal, with a 2.3-fold difference in maximum EGFP signal between the two lines. As we discuss below, the distinct time courses of EGFP expression for the two serosal lines suggest that the respective enhancer traps reflect different features of the serosa.

### Dynamic cellular morphology arises within the dorsal-central serosa

**Figure 2:**
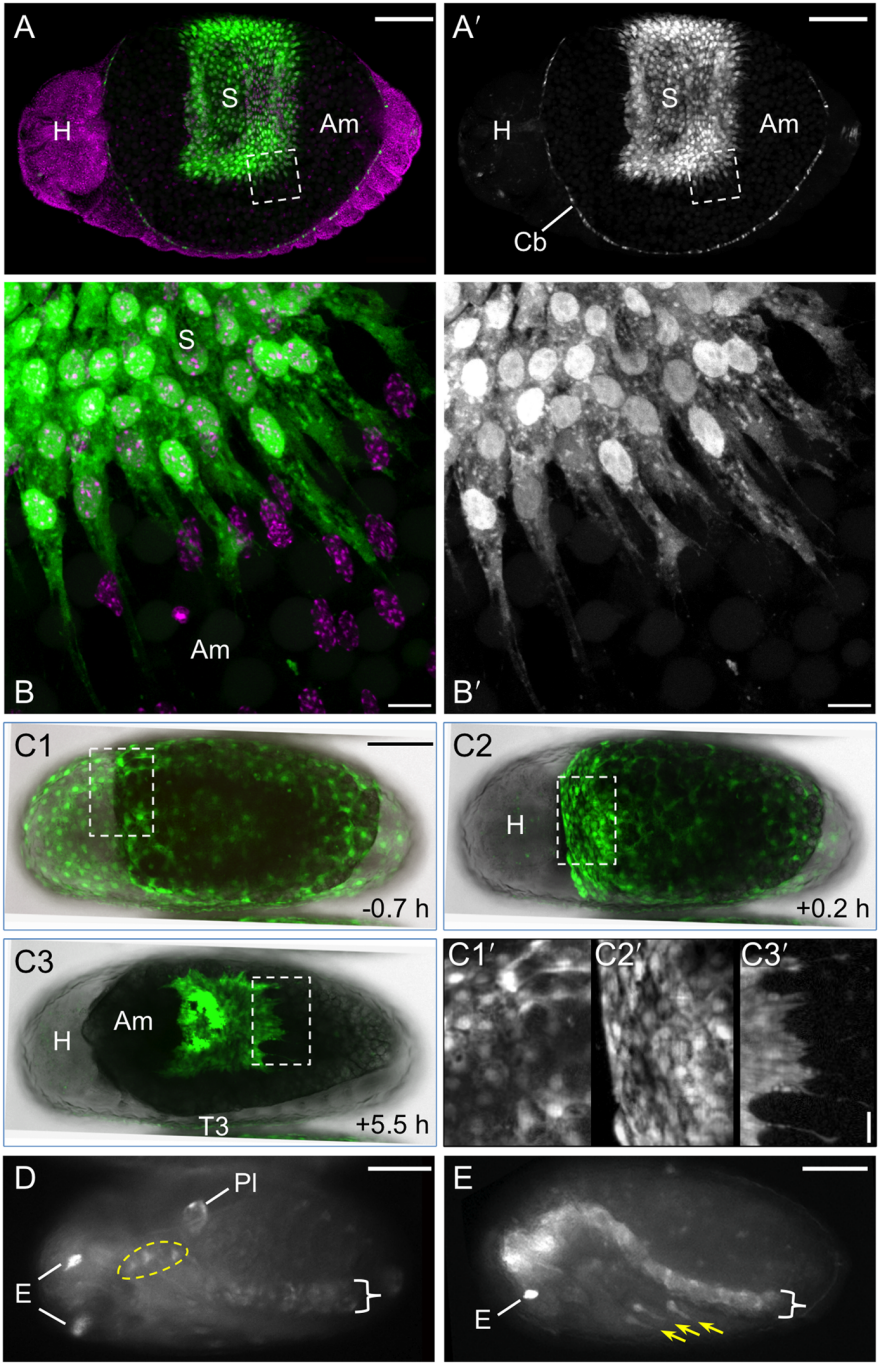
Specific expression features for the lines G12424 and KT650. Views are dorsal (A–C), ventral-lateral (D), or lateral (E), with anterior left. (**A**–**C**) In both lines EGFP (green; white in single-channel images) fills serosal cells, showing the nucleus and cytoplasmic protrusions or overall cell shapes (A–B: G12424×G04609-mesodermal heterozygote: EGFP in the cardioblasts (Cb) is from the G04609 line, described below; C: KT650). EGFP expression throughout the serosa (S) clearly demarcates the tissue boundary with the unlabeled amnion (Am). In fixed specimens (A–B) a nuclear counterstain in shown is magenta. In live imaging panels (C1–C3), whole mount views merge the EGFP and transmitted light channels: the opaque yolk appears black and the embryonic tissue is light grey. Time stamps in C1–C3 are relative to serosal rupture (at 24°C). Inset images C1′–C3′ show details of cell shapes: orderly polygons over the embryo (left) and more irregular apical areas over the yolk (right) in the pre-rupture serosa (C1′); at the anterior edge of the serosa during early contraction (C2′); and at the posterior edge as the serosa begins to degenerate, with trailing cytoplasmic extensions (C3′). See also Movie S1. (**D**–**E**) In addition to serosal expression, both lines exhibit late embryonic expression. EGFP in the eyes (E) and ventral nerve cord (curly brackets) is the result of the 3xP3 promoter within the transposon and is not line specific. Line G12424 (panel D) exhibits EGFP in the pleuropodia (Pl) and a small proximal region of the legs (dashed yellow outline encompasses T1–T3), while line KT650 (panel E) has expression in the distal portion of the legs (yellow arrows) and strongly in the central nervous system, including the brain and at heightened levels in the ventral nerve cord compared to 3xP3 expression alone. Scale bars are 100 µm for all whole mount images; for the inset images, scale bars are 10 µm (B) and 20 µm (C1′-C3′).

Initially, EGFP expression is quite homogeneous throughout the serosal cells. The thicker, central portion of the cell body associated with the nucleus is brightest, with the thinner, peripheral cytoplasm showing a general, diffuse EGFP signal as a fuzzy halo around the nucleus (Fig. 1C3′; higher resolution shown in Figure 2, below). However, during late germband extension, the serosal cells acquire a new morphological feature, with bright streaks of EGFP appearing to connect neighboring nuclei as vertices of irregular polygons of EGFP across the tissue (Fig. 1C4′). These streaks are highly dynamic, defining different polygon shapes over time as they connect difference vertices. Although often the EGFP in the nuclear region is continuous with these streaks, not all vertices correspond to a serosal nucleus, and not all serosal nuclei are connected in a polygon structure at a given time. These dynamic changes in EGFP localization occur despite the fact that overall the serosal cells do not actually move or change neighbors within the tissue. Once these streaks of EGFP appear, they persist throughout the period when the serosal cells are a squamous monolayer, prior to tissue folding and thickening to form the dorsal organ [16]. Although the streaks occur very broadly throughout the tissue, they seem to be more prevalent on the dorsal and lateral surfaces over the yolk than on the more ventral surface over the amnion and embryo. Given that the serosal nuclei do not themselves move and are not always associated with the dynamic streaks, we cannot preclude the possibility that this EGFP expression occurs below the serosa, possibly in the cortical region of the yolk itself. However, as we detect no EGFP in the visible yolk when the ruptured serosa is contracting (see above) – at which time the EGFP streaks still persist within the moving serosal tissue – we nonetheless attribute this unusual feature to the serosa itself. These EGFP streaks also occur in the KT650 line, in this case from the onset of expression throughout the serosa, consistent with the later stage at which serosal EGFP develops with this line and indicating that the EGFP streaks reflect a stage specific alteration within the serosa.

Throughout the stages of serosal tissue development, EGFP fully illuminates the cells, including both the nucleus and full cytoplasmic volume (Fig. 2A–C). In the monolayered epithelium, this makes it possible to distinguish the cortical regions and hence the polygonal shape of serosal cells amongst their neighbors, as does the fact that EGFP intensity varies across cells (Figs. 2C1,C2, C1′,C2′; Movie S1). As the serosa compacts and begins to degenerate, even highly attenuated cytoplasmic protrusions at the trailing edge are visible in both fixed and live specimens (Fig. 2B,B′,C3,C3′; Movie S1).

### Non-serosal expression domains in late embryogenesis

Although both G12424 and KT650 are predominantly serosal markers, a few embryonic domains also express EGFP at late stages. At the retracted germband stage, G12424 shows expression in the pleuropodia on the first abdominal segment while KT650 expression includes the distal tips of the legs, and these domains persist through post dorsal closure stages (Fig. 2D–E). After serosal rupture, leg expression occurs in G12424 in a small proximal domain (Fig. 2D). The 3xP3 promoter within the transposon drives expression in the eyes and ventral nerve cord in all lines, beginning after dorsal closure (*e.g.*, Fig. 2D). However, KT650 expression does seem to include a central nervous system domain encompassing both the brain and the ventral nerve cord, as this expression arises earlier, during dorsal closure, and occurs at a stronger level (Fig. 2E).

### Mapping of the G12424 and KT650 insertion sites and preliminary evaluation of candidate ‘target’ genes

**Figure 3:**
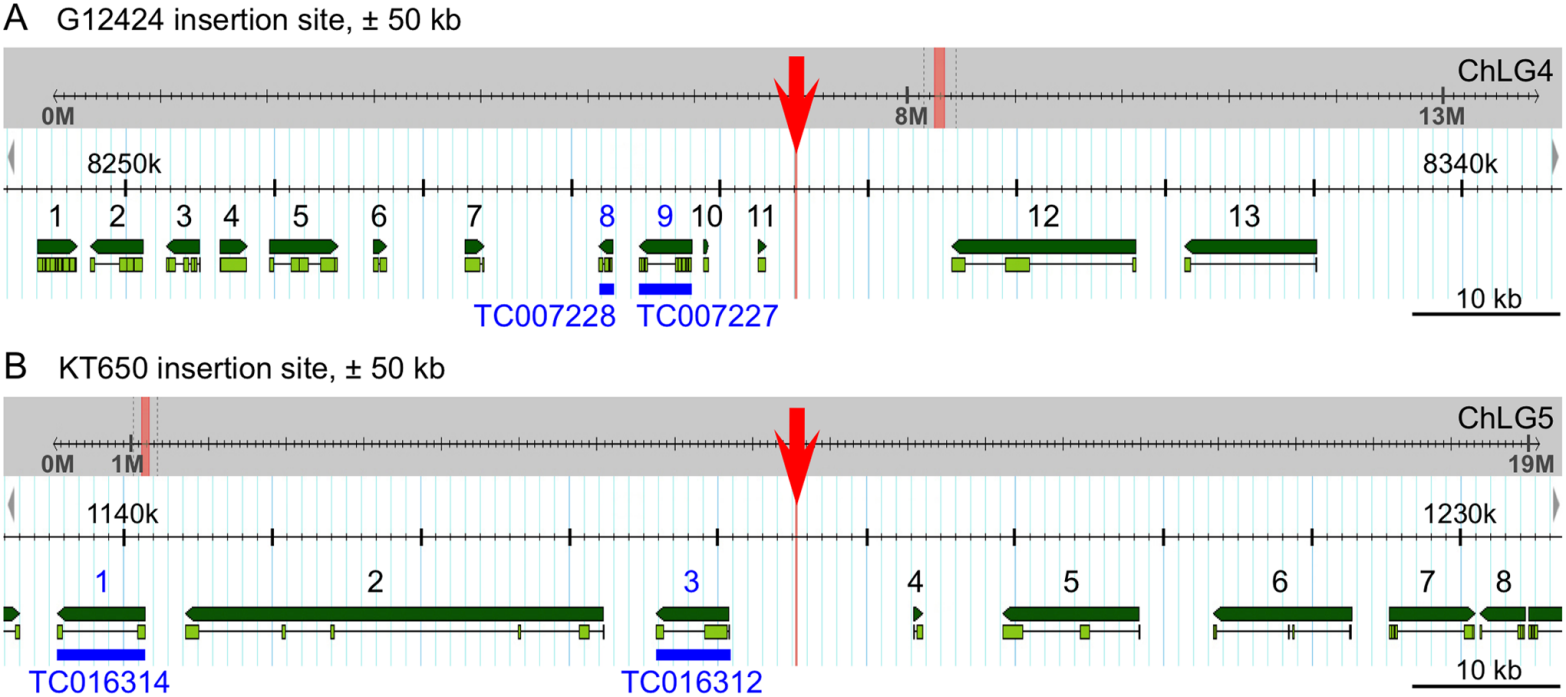
Genomic mapping of the serosal lines G12424 (A) and KT650 (B). Genome browser views (version Tcas_3.0) show the insertion site (red) within the context of the entire chromosomal linkage group (ChLG, grey background) and enlarged for the region ±50 kb from the insertion site (white background). Predicted genes (Tcas_3.0 official gene set) within this region are numbered and shown in green (dark green: entire gene, light green: exons); see Tables 2 and 3 for details of specific genes. Candidate genes inspected by *in situ* hybridization are labeled in blue.

Both the G12424 and KT650 lines specifically have serosal EGFP expression. To begin to determine which gene’s enhancer(s) had been trapped, we mapped the insertion sites and examined the genomic neighborhood for candidate target genes (see Methods). The TTAA target site, at which *piggyBac* preferentially integrates [22], is intergenic in both lines (Fig. 3A–B). G12424 integration occurred on chromosomal linkage group 4 (genome version Tcas_3.0, ChLG4: 8,295,121.24; Assembly 4.0, ChLG4∶7,103,734.37). In line KT650, the transposon is on chromosome 5 (Tcas_3.0, ChLG5∶1,185,266.69; Assembly 4.0, ChLG5∶860,696.99). In both lines, the transposons inserted upstream of and in the opposite reading direction to the nearest candidate gene tested (TC007227 and TC016312, respectively: see Fig. 3).

In the case of line G12424, the integration site was already mapped within the context of the original insertion screen [5], and our analysis confirms this location. The original analysis also identified the *Tribolium* orthologue of the *Synaptotagmin 1* gene (*Tc-Syt 1*, gene number TC007227 in the Tcas_3.0 official gene set) as the nearest candidate gene whose enhancer may have been trapped (Fig. 3A). However, attempted *in situ* hybridization with specific probes for *Tc-Syt 1* and its downstream neighbor, *Tc-Trf* (TC007228: see Table 2 for details) yielded no specific staining in the serosa at any developmental stage. For *Tc-Syt 1*, we obtained a ubiquitous stain in the yolk but not in the cells at blastodermal stages, and faint expression in the brain and ventral nerve cord occurred in older stages (retracted germband through post-dorsal closure), where the latter expression is consistent with a conserved role of this orthologue in synaptic vesicle trafficking [23], [24]. For *Tc-Trf*, we could not detect localized transcript at any developmental stage.

**Table 2:** Candidate target genes in the vicinity (±50 kb) of the G12424 insertion, as numbered in Fig. 3A.

#	Gene ID^a^	Orthology name andFlyBase CG#^d^	*Drosophila* gene descriptions, including stages of peak expression (PE) and embryonic *in situ* (eISH) expression notes (citations in Methods)
1	TC008244 (au2.g6126.t1; au3.g5375.t1)	No orthologue;best hit: *goliath*(*gol*; CG2679)	Zinc-finger transcription factor; role in mesoderm development; PE: 6–24 h embryonic and all later stages; eISH: visceral and pharyngeal muscles, stages 11–16
2	TC007230 (au2.g6127.t1; au3.g5376.t1)	–	No homologues in *Drosophila*. Eleven similar genes in *Tribolium.*
3	TC007229 (au2.g6129.t1; au3.g5378.t1)	*Ef1y* (CG11901)	Translation elongation activity; PE: 0–12 h embryonic and early larval; eISH: maternal and ubiquitous, stages 1–16
4	TC008245 (au2.g6130.t1; au3.g5379.t1)	CG3702	Transmembrane protein; larval lethal; PE: 0–18 h embryonic and all later stages; eISH: maternal and ubiquitous, stages 1–16
5	TC008246 (au2.g6131.t1; au3.g5380.t1)	*Glycogen* *phosphorylase*(*Gly*; CG7254)	Innate immune response and glycogen catabolism; PE: 0–6 and 12–24 embryonic and all later stages; eISH: rapidly degraded, ubiquitous maternal expression, later somatic muscles, stages 1–6 and 13–16
6	TC008247^b^ (au2.g6132.t1; au3.g5381.t1)	–	No homologues in *Drosophila* or *Tribolium*.
7	TC008248^b^ (au2.g6132.t1; au3.g5381.t1)	–	No homologues in *Drosophila* or *Tribolium*.
8	TC007228^c^ (au2.g6133.t1; au3.g5382.t1)	*TBP-related* *factor* (*Trf*;CG7562)	TFIIA-class transcription factor; PE: 0–6 h embryonic; eISH: maternal and ubiquitous, stages 1–12
9	TC007227^c^ (au2.g6135.t1; au3.g5384.t1)	*Synaptotagmin* *1* (*Syt 1*;CG3139)	Calcium binding; neurotransmitter transport function; PE: 12–24 h embryonic and all later stages; eISH: central nervous system, stages 13–16
10	TC008249 (–; TC008249)	–	No homologues in *Drosophila* or *Tribolium*.
11	TC008250 (au2.g6136.t1; au3.g5385.t1)	–	No homologues in *Drosophila* or *Tribolium*.
12	TC007226 (au2.g6137.t1; au3.g5386.t1)	*kekkon-3*(*kek3*; CG4192)	Immunoglobulin-like domain; PE: 12–24 h embryonic and all later stages, including adult brain; eISH: no staining detected (with EST IP22191), stages 1–16
13	TC007225 (–; TC007225)	–	No homologues in *Drosophila* or *Tribolium*.

a Genes are identified by protein prediction models from Tcas_3.0 (official gene set (OGS): TC#, shown in Fig. 3A), AUGUSTUS2 (au2, from July 2012), and AUGUSTUS3 (au3, from September 2013).

b Indicates a gene with multiple OGS identifiers but a single Augustus model.

c Indicates genes for which *in situ* hybridization was tested.

d Based on reciprocal BLAST identification of the probable *Drosophila melanogaster* orthologue, or non-orthologous best hit, within the *Tribolium* genome browser.

However, there are a number of other predicted genes within ±50 kb of the insertion site (Fig. 3A). Based on information for the *Drosophila melanogaster* orthologue or nearest homologue (see Methods), we compiled a list of potentially relevant expression or molecular function information for these candidates (Table 2). As the extraembryonic tissue component in the fruit fly is secondarily reduced [13], it is difficult to predict which features would be indicative of a good candidate gene. For example, some aspects of *Tribolium* serosal function, such as cuticle secretion, involve genes active at the pupal stage in *Drosophila* for the same purpose [18], [25], [26]. As our primary aim here is to characterize the enhancer trap lines, we provide this preliminary information for future work on identifying the relevant target gene or for a direct search for the trapped enhancer(s).

Similarly, *in situ* hybridization for two genes near to the KT650 insertion (Fig. 3B: blue annotations) did not reveal localized transcripts at any stage. There was weak, ubiquitous expression above background levels in the embryo, but the yolk and extraembryonic tissues were unstained. In Table 3 we have summarized potentially pertinent features of all genes within ±50 kb of this insertion for future reference.

**Table 3:** Candidate target genes in the vicinity (±50 kb) of the KT650 insertion, as numbered in Fig. 3B.

#	Gene ID^a^	Orthology name and FlyBase CG#^d^	*Drosophila* gene descriptions, including stages of peak expression (PE) and embryonic *in situ* (eISH) expression notes (citations in Methods)
1	TC016314^c^ (–; au3.g5845.t1)	CG7049	Sulphatase enzyme; PE: 0–6 embryonic and pupal, expressed in many adult organs; eISH: no entry in database
2	TC016313^b^ (au2.g6668.t1, au2.g6669.t1 (partial); au3.g5844.t1, au3.g5843.t1 (partial))	CG11905	Unknown molecular function; PE: pupal stages; eISH: dorsal and head epidermis, tracheal system, and posterior spiracles, stages 11–16
3	TC016312^b^^,^ c (–; au3.g5843.t1 (partial))	CG11905	Unknown molecular function; PE: pupal stages; eISH: dorsal and head epidermis, tracheal system, and posterior spiracles, stages 11–16
4	TC016319 (au2.g6670.t1; au3.g5842.t1)	–	No homologues in *Drosophila* or *Tribolium*.
5	TC016311 (au2.g6671.t1; au3.g5841.t1)	*Cuticular protein 73D*(*Cpr73D*; CG9665)	Structural constituent of chitin-based cuticle; PE: late pupal stages; eISH: restricted regions of the ventral epidermis, stages 13–16
6	TC016310 (au2.g6672.t1; au3.g5840.t1)	*Odorant-binding protein 73a* (*Obp73a*)	Pheromone binding; PE: late pupal stages; eISH: no entry in database
7	TC016320 (au2.g6673.t1; au3.g5839.t1)	CG4729	Phospholipid/glycerol acyltransferase; PE: 12–18 h embryonic and all later stages in all tissues; eISH: maternal and ubiquitous during stages 1–10, later in the endoderm, midgut, procrystal cell, ring gland, oenocytes, and posterior spiracle during stages 9–16
8	TC016309 (au2.g6674.t1; au3.g5838)	*Gonadal* (*gdl*; CG33756)	Gonadogenesis; PE: pupal and adult male; eISH: no entry in database

a Genes are identified by protein prediction models from Tcas_3.0 (official gene set (OGS): TC#, shown in Fig. 3B), Augustus2 (au2, from July 2012), and Augustus3 (au3, from September 2013).

b Indicates a gene with multiple OGS identifiers but a single Augustus model.

c Indicates genes for which *in situ* hybridization was tested.

d Based on reciprocal BLAST identification of the probable *Drosophila melanogaster* orthologue, or non-orthologous best hit, within the *Tribolium* genome browser.

### The G04609 line labels mesodermal and sensory domains as well as the serosa

We selected the line G04609 for detailed examination as the GEKU database information suggested dorsal embryonic expression, a useful feature for examining the process of dorsal closure [16]. Our analyses are the same as for the serosal lines, using a combination of live imaging on different microscopes and anatomical evaluation of fixed specimens.

**Figure 4:**
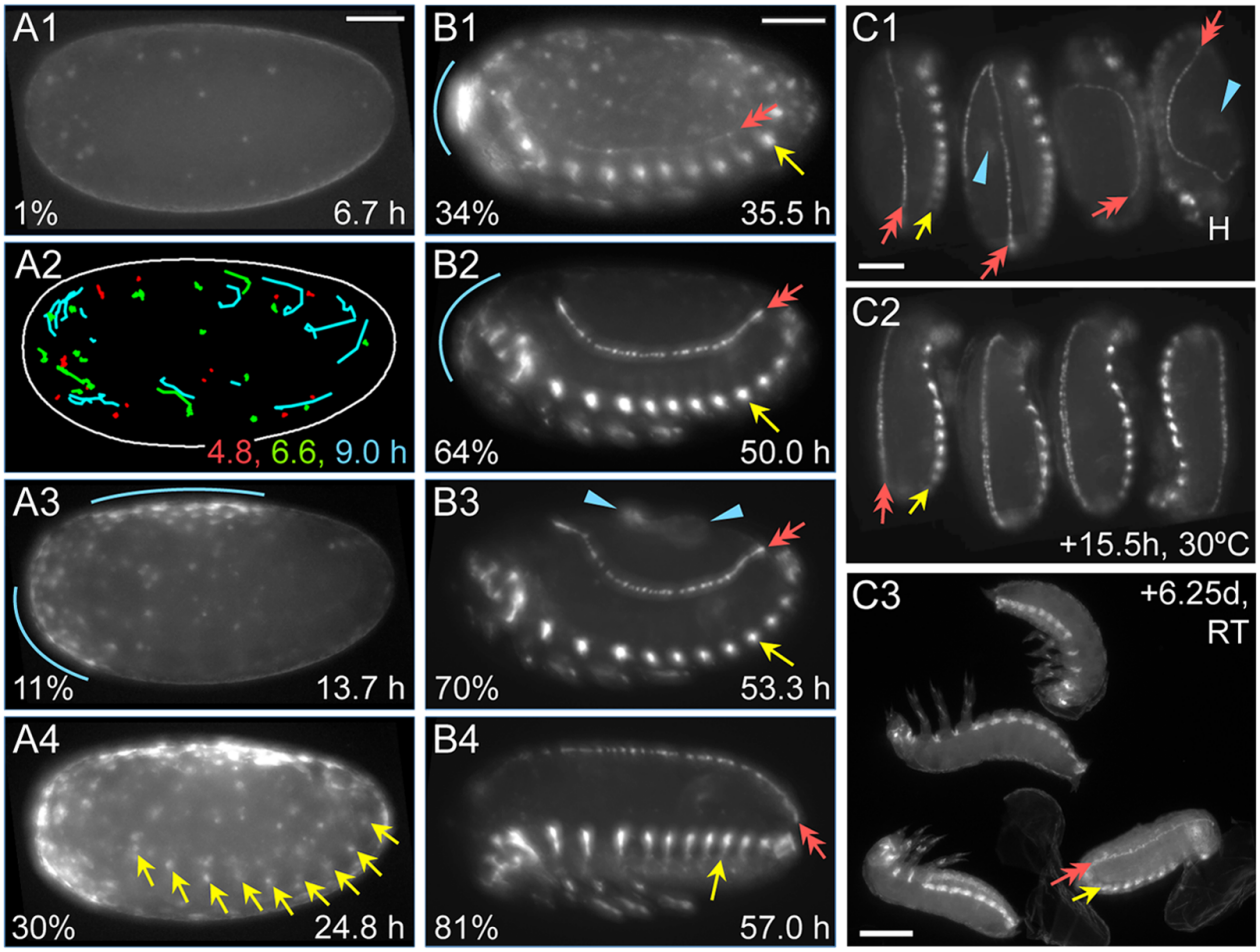
EGFP expression in the G04609 line across embryogenesis. Micrographs labeled with the same letter are of the same embryo(s). Views are in lateral (A–B) or dorsal-lateral (C) aspect, with anterior left (A–B) or up (C, except left-most embryo, where the head is labeled: H). (**A**) Onset of expression from blastoderm through germband extension stages (4.8–24.8 hAEL, n = 11), including in the yolk and dorsal-anterior serosa. (**B**) Increasing EGFP signal through the completion of dorsal closure (17.9–64.2 hAEL, n = 7), in lateral segmental muscle blocks, distal leg regions, and the cardioblast cell row. See also Movie S2. (**C**) Overview of expression from dorsal closure through hatching of the larvae (n = 11). Images A1–A2 show expression in yolk globules, where the schematic in A2 tracks all globules visible at each of the three ages indicated for the entire duration that a given globule is visible. In all subsequent panels, colored annotations indicate: EGFP expression in the serosa (blue, arcs and arrowheads: A3,B1–B3,C1), in the segmental muscle blocks (yellow arrows: A4,B1–B4,C1–C3), and in the cardioblast cell row (red double-headed arrows: B1–B4,C1–C3). In B1–B4, only the segmental muscle block on abdominal segment 5 is indicated. Percentage values (A–B) are normalized EGFP signal intensity values relative to the maximum intensity achieved during embryogenesis. Time stamps show embryo minimum age (A–B, at 30°C) or elapsed time from start of filming (C, 30°C then room temperature). All scale bars, shown in the first panels only, are 100 µm except for 200 µm in C3.

As with the serosal lines, for G04609 all early eggs (n = 11) show dynamic, round globules of weak EGFP throughout the yolk (Fig. 4A1), from the onset of filming at the blastoderm stage at least through to the early germband extension stage, after which this signal is lost relative to the onset of additional, tissue-specific expression domains. On average the globules are only briefly visible in the cortex (for 43 minutes) before sinking back into the yolk. Within the cortical plane, they only move in short, random walks (24 µm, or 4% egg length) until wholesale displacement occurs at the time of primitive pit formation through serosal window closure (n = 65 yolk globules from three eggs; Fig. 4A2: early red compared to later green and cyan tracks).

Early, localized G04609 EGFP is actually within two distinct regions of the serosa: just posterior-ventral to the underlying embryo’s head, and a dorsal region spanning 50–75% egg length (Fig. 4A3). This expression is first weakly detected during early germband extension, at 14.0±0.9 hAEL, or two hours after serosal window closure. Serosal expression then expands and strengthens throughout the tissue through maximum germband extension, although in all cases the dorsal-anterior bias is still discernible (Fig. 4A4). However, these early expression domains are weak both relatively (only 1–30% of maximum EGFP intensity achieved in this line) and absolutely (comparable EGFP intensity values to line KT650 during Movie S1: Fig. 1H). Serosal EGFP persists at these weak levels throughout the life of this tissue, retaining stronger expression in the anterior (Fig. 4B1–B3,C1: blue annotations).

**Figure 5:**
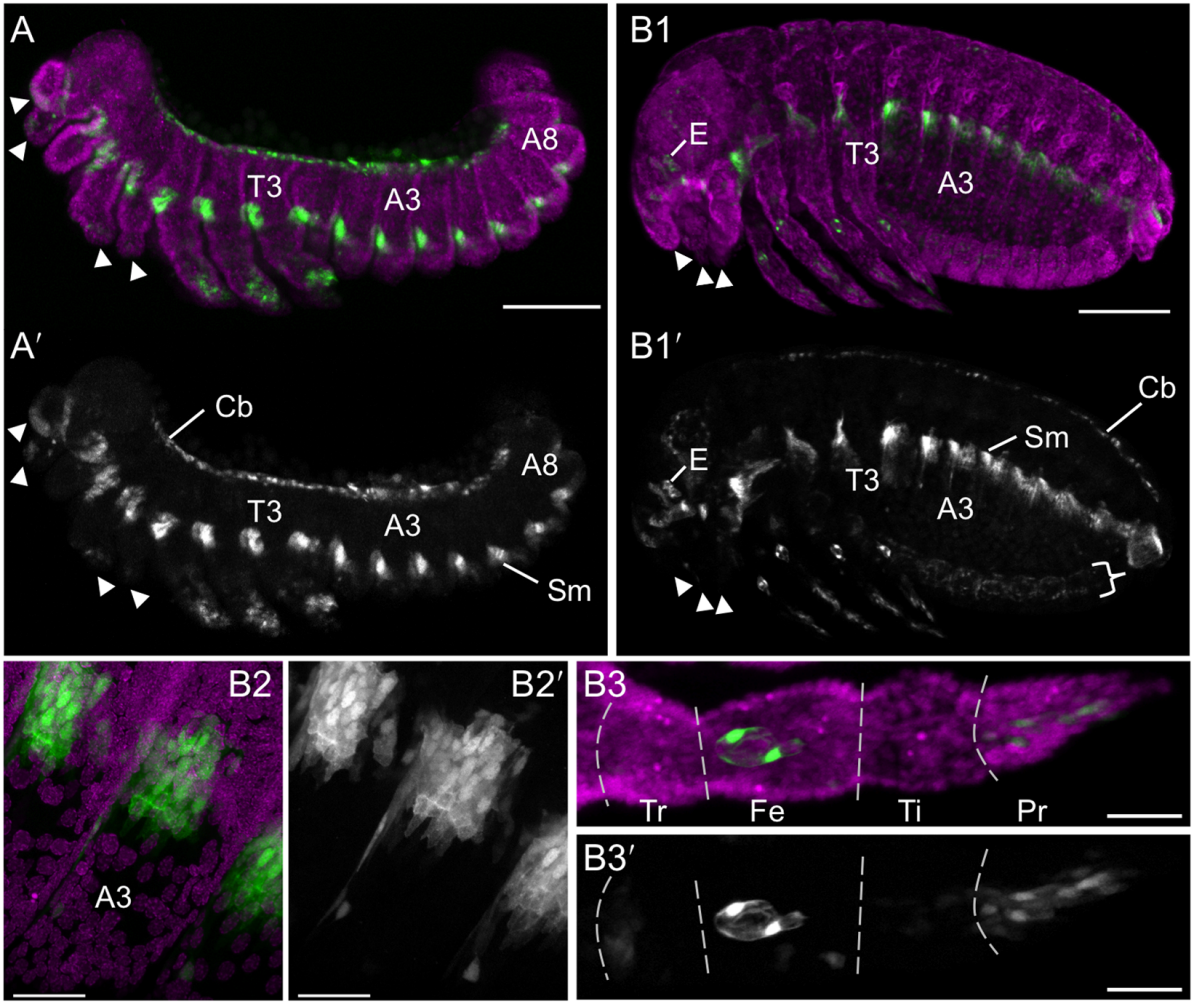
Expression in the line G04609: muscle and leg sensory cell anatomical details. For the retracted germband (**A**) and post-dorsal closure (**B**) stages, representative embryos are shown with G04609-mesodermal×G12424-serosal heterozygote EGFP expression (green; white in single-channel images) and a nuclear counterstain (magenta). The serosa and dorsal yolk have been removed in image A. G04609 muscle EGFP persists in lateral segmental blocks (A,B1,B2). Meanwhile, expression in the appendages is dynamic. The distal tips of the labral, antennal, maxillary, and labial segments (but not the mandibulary segment) initially express EGFP, but this is subsequently lost (compare arrowheads in A and B1). In the legs, distal expression is initially broad (A) and later refines to a subset of putative sensory structures (B1,B3): a small, anterior-proximal patch in the trochanter, a circular structure and single anterior-distal cell within the femur, and cells throughout the pretarsus (shown for the T2 leg). The expression in the trochanter and single labeled cell in the femur are superficial and likely represent sensory cells within the ventral epidermis, such as campaniform sensillae [55]. The circular structure lies in the center of the femur’s diameter and is anatomically consistent with identification as the chordotonal organ [56]. The expression in the pretarsus is distally superficial but innervates the leg more proximally and also likely represents sensory structures. Lastly, EGFP expression also occurs weakly throughout the ventral nerve cord at post-dorsal closure stages, due to the 3xP3 enhancer in the transposon (B1′: curly bracket). All views are maximum intensity projections in lateral aspect, with anterior left or proximal left (B3 only). Abbreviations: A(x), abdominal segment (x); Cb, cardioblasts; E, eye; Fe, femur; Pr, pretarsus; Sm, segmental muscle blocks; T(x), thoracic segment (x); Ti, tibiotarsus; Tr, trochanter. Scale bars are 100 µm (A,B1) and 20 µm (B2,B3).

**Figure 6:**
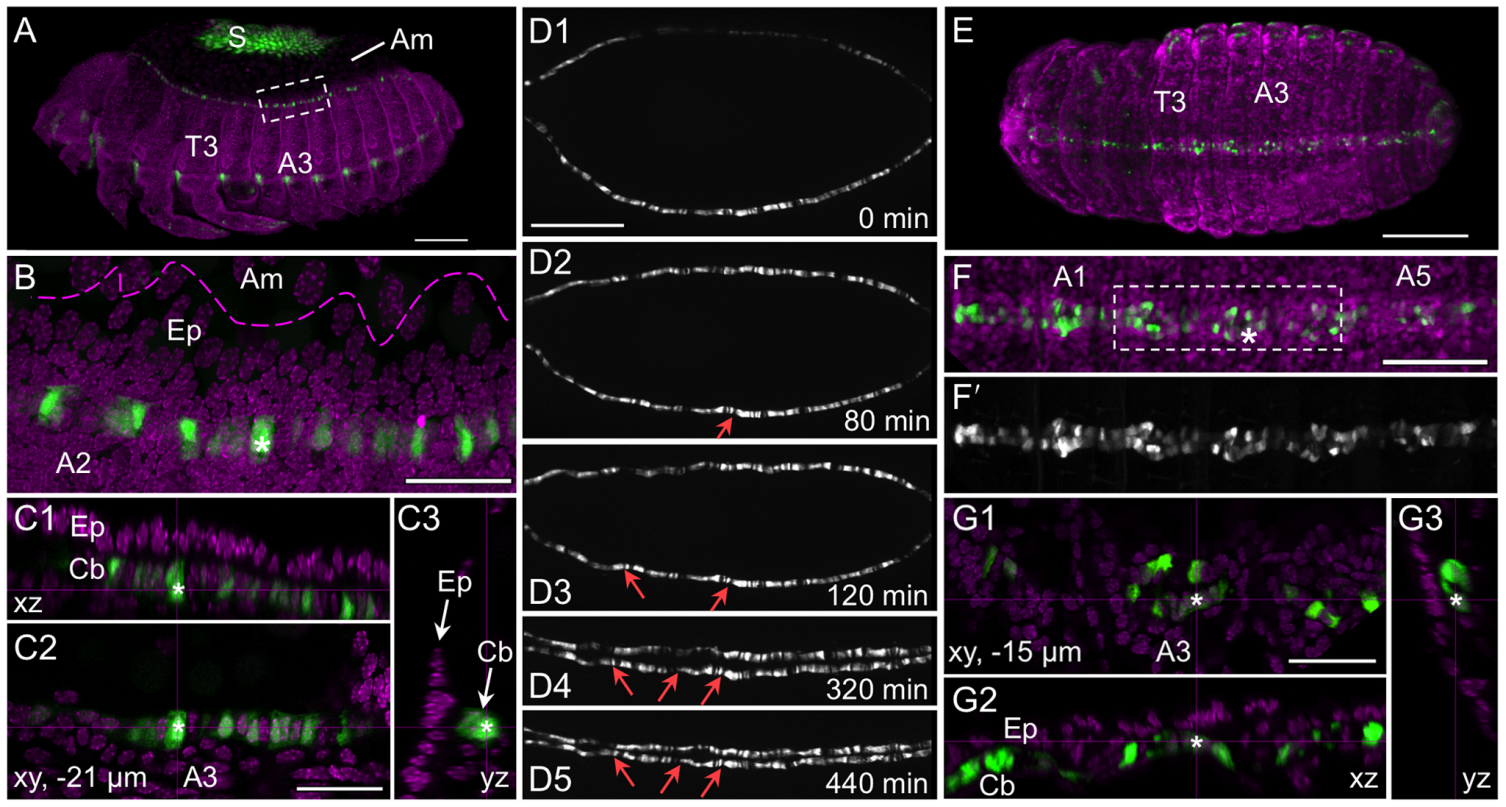
Mesodermal expression in the line G04609: heart anatomical details. For the retracted germband (**A**–**C**), dorsal closure (**D**), and post-dorsal closure (**E**–**G**) stages, representative embryos are shown with G04609-mesodermal×G12424-serosal heterozygote (A–C,E–G) or G04609 homozygote (D) EGFP expression (green; white in single-channel images) and a nuclear counterstain in fixed specimens (magenta). The serosa is visible in panel A only. Throughout these stages, G04609 heart EGFP is stably expressed in a subset of cardioblast cells, near to the dorsal epidermis-amnion boundary (B: dashed magenta line approximation, based on [16]) and directly below the epidermis (C1–C3,G1–G3). As heart formation progresses during dorsal closure, small bends form in the cardioblast cell row (D2–D5: red arrows), reflecting the overall scalloped, or wavy, geometry of dorsal closure (compare with shape of dashed line in B). Note that in D1 the upper cell row is not yet fully in view due to the slightly oblique angle. Views are lateral (A–C) or dorsal (D–G), with anterior left. All views are maximum intensity projections except C1–C3 and G1–G3 provide orthogonal views of a single plane (depth below surface is indicated in the xy panel: C2,G1). Boxed regions in A and F correspond to panels B and G, respectively. The focal point for orthogonal views is indicated by a white asterisk (B–C,F–G). Time stamps in D1–D5 show elapsed time from start of filming (at 24°C). Abbreviations: A(x), abdominal segment (x); Am, Amnion; Cb, cardioblasts; Ep, epidermis; S, serosa; T(x), thoracic segment (x). Scale bars are 100 µm (A,D,E), 20 µm (B,C,G), and 50 µm (F).

As development proceeds, EGFP signal arises in multiple domains. Among these, expression is first detected in lateral, segmentally repeated blocks of muscle during germband extension, beginning 16.4±0.9 hAEL (n = 8; embryos with particularly strong overlying serosal EGFP were excluded from this measurement; Fig. 4A4: yellow arrows). Subsequent expression also develops in the cardioblast cell row (presumptive heart), beginning about midway through germband retraction (Fig. 4B1: red arrow). Expression in both of these domains strengthens through the end of embryogenesis and then persists in the hatched larva (Figs. 4B–C, 5, 6). Additionally, EGFP expression occurs in the distal region of the appendages, in a dynamic fashion that refines over time and likely comprises a subset of sensory cell structures (Figs. 4B1–B4, 5A,B1,B3). While the lateral muscle block regions seem to be fully labeled by EGFP, in a segmentally iterated and characteristic shape (Fig. 5B2), heart expression does not occur in all cardioblasts (Fig. 6A–C) although EGFP expression appears to be stably on or off in a given cell (Fig. 6D1–D5).

### Evaluation of the genomic context suggests that G04609 expression is consistent with partial trapping of *Tc-midline* enhancer(s)

**Figure 7:**
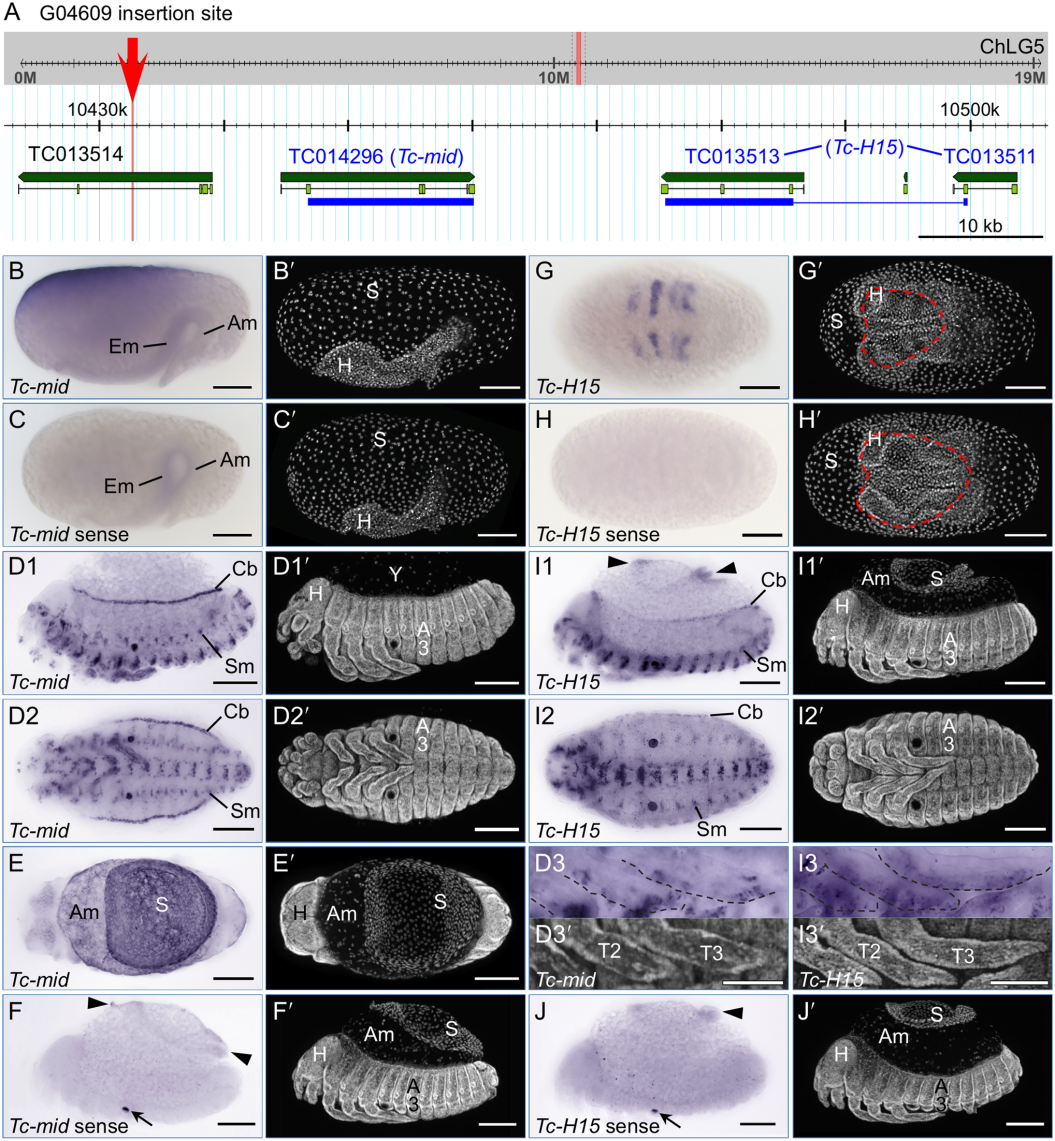
Line G04609 enhancer trapping evaluation: genomic mapping and expression of neighboring genes (*midline* and *H15*). (**A**) Genome browser views (version Tcas_3.0) show the insertion site (red) within the context of the entire chromosomal linkage group (ChLG, grey background) and enlarged for the region 75 kb 3′ to the insertion site (white background). Predicted genes (Tcas_3.0 official gene set) within this region are labeled and shown in green (dark green: entire gene, light green: exons). Blue bars span the exons used as cDNA template for *in situ* hybridization. In the case of *H15*, this spanned the TC013511 and TC013513 gene models, omitting the linking region marked by the thin line. In newer models, *Tc-mid* is represented by au2.g7325.t1 or au3.g6464.y1; *Tc-H15* is au2.g7328.t1 or au3.g6465.t1 (explained in Table 2). (**B**–**J**) *In situ* hybridization for the nearby candidate genes *Tc-mid* (B–F) and *Tc-H15* (G–J), for the early stage of serosal window closure (B,C,G,H), and for the later stages of the retracted germband and serosal contraction/dorsal organ stage (D–F,I–J). See the main text for descriptions of these expression domains. All images labeled with the same letter are of the same embryo (except D3). Letter-prime images show the nuclear counterstain. Views are lateral (B,C,D1,F,I1,J), ventral (D2,D3,G,H,I2,I3), or dorsal (E), with anterior left. In D, the serosa has been removed from the embryo, exposing the yolk (Y). Annotations: red dashed line, serosal window outline (G,H); arrowheads, the outer, folded parts of the contracting serosa (F,I1,J). Additionally, dashed outlines in inset images (D3,I3) outline the anterior margin of the legs. Sense strand controls (C,F,H,J) show increased background levels in the folded, thickened outer regions of the contracting serosa (F,J), and non-specific stain in the pleuropodia (arrows in F,J). Abbreviations as in previous figures, and Em, embryo. Scale bars are 100 µm, except for 50 µm for the inset images (D3,I3).

We mapped the G04609 insertion to chromosome 5 (Tcas_3.0, ChLG5∶10,432,666.69; Assembly 4.0, ChLG5: 8,292,056.59; Fig. 7A), within the intron of the hypothetical gene TC013514. However, there is a 696 bp stretch between the TTAA target site and the 3′ end of the piggyBac transposon. This is a genuine, unique sequence stretch within the genome of the G04609 line, as confirmed by two independent inverse PCR preparations as well as direct PCR amplification with specific primers to this region. However, it is comprised of a jumble of discontinuous, shorter fragments that map to various other locations in the genome, and we could not amplify this stretch in wild type. We therefore conclude that this reflects an unusually messy transposon insertion event.

Despite this complication, we are confident of this mapping position for two reasons. Firstly, the unique, reproducible inverse PCR fragment included 331 bp that specifically map adjacent to this genomic location. Secondly, the insertion site is near two genes whose expression patterns are similar to the enhancer trap EGFP expression: the *Tribolium* orthologues of *midline* (*Tc-mid*) and *H15* (*Tc-H15*).

Both genes are T-box transcription factors, a class of regulators with conserved roles across flies and vertebrates in mesodermal tissues, and particularly in heart development [27]. To ascertain whether the *Tribolium* orthologues of these genes are expressed in a comparable fashion, we examined both by *in situ* hybridization (Fig. 7B–J). In the case of *Tc-H15*, this augments a previous, brief study that used an *in situ* probe detecting a subset of the same region of the transcript ([28]; see Table 4 for probe details). The endogenous transcript expression patterns are indeed consistent with a conserved developmental role and with the G04609 EGFP profile, as we detected expression in the cardioblasts and in segmentally repeated lateral blocks. However, the complete expression profiles of these genes are distinct from one another and do not precisely recapitulate G04609 expression.

**Table 4:** List of primers used for generating *in situ* hybridization templates.

GEKU line	Gene	Forward or sense primer (5′ to 3′)^b^	Reverse or antisense primer (5′ to 3′)^c^	Amplicon size (bp)
G12424	TC007227(*Tc-Syt 1*)	CAGTTTCAAGTGGAGGCACA	TGCACTTCTTGATGCTGGTC	993
G12424	TC007228(*Tc-Trf*)	GTTGAATAGCCCTGCACGTT	TCCAGCGATTCCTTAATTCC	639
KT650	TC016312	GGAACAAGTGGCAACGATTT	TTGACGTCCAGTGGGTTGTA	658
KT650	TC016314	GTGGCTGTCACCTCAACAGA	AGATATTGGCCCAGTGTTCG	639
G04609	TC014296(*Tc-mid*)	AGTTCAACGAATTGGGAACG	TCAGAAACAACTGCGACCTG	699
G04609	TC013513+ TC013511^a^(*Tc-H15*)	ACGATCTTGGCACGGAAATG	TAGATTTGCTGGCTTGGGGA	787
–	*EGFP*	ACGTAAACGGCCACAAGTTC	CTTGTACAGCTCGTCCATGC	669
–	T7 RNA promoteruniversal primers	GAGAATTCTAATACGACTCACTATAGGGCCGCGG	AGGGATCCTAATACGACTCACTATAGGGCCCGGGGC	–

a In the *Tribolium* official gene set, the coding sequence for *Tc-H15* is incorrectly split between these two gene models: see Fig. 6A.

b Forward primers also included the 5′ adapter sequence 5′-GGCCGCGG-3′ for subsequent amplification with the T7 promoter universal primer for sense strand transcription (adapter not shown in table).

c Reverse primers also included the 3′ adapter sequence 5′-CCCGGGGC-3′ for subsequent amplification with the T7 promoter universal primer for antisense strand transcription (adapter not shown in table).

*Tc-mid* is expressed in early development in a localized domain that partially spans one egg pole and part of the longitudinal surface. This is primarily seen at the syncytial to uniform blastoderm stages, but inter-embryo differences in scope and exact location of the domain suggest a highly dynamic, or variable, pattern. In most cases, all detectable transcript is gone before the differentiated blastoderm stage. However, based on less frequently observed expression during the serosal window stage, this expression may, in part, prefigure an anterior-dorsal portion of the serosa (Fig. 7B). No transcript is detected in the embryo above background levels (compare Fig. 7B and 7C) until the much later stages of heart and muscle differentiation (Fig. 7D1–D2). Also, new expression throughout the extraembryonic domain –stronger in the serosa than in the amnion – occurs at the time of serosal contraction to form the dorsal organ (Fig. 7E). In contrast, *Tc-H15* is expressed in segmental stripes from the differentiated blastoderm stage through germband extension (Fig. 7G) and is not expressed in the extraembryonic tissue above background levels at any stage (Fig. 7G and I1, compared to 7J). *Tc-mid* is expressed more strongly in the cardioblasts and segmental muscles than *Tc-H15* (Fig. 7D1–D2 compared to 7I1–I2). Although both genes also show segmentally repeated expression patterns along the ventral midline, they are not identical, with *Tc-H15* slightly more medial (Fig. 7D2,I2). Lastly, although both genes are also expressed in the legs, late *Tc-mid* expression is restricted to a few discrete domains including a small circular patch and the distal tip, while *Tc-H15* is more broadly expressed throughout the anterior margin of the appendage (Fig. 7D3,I3). Taken together we find that the G04609 EGFP expression is more similar to that of *Tc-mid*, and discuss the strength of this conclusion below.

## Discussion

Here we discuss four aspects of the EGFP expression in the enhancer trap lines: (i) characteristics of the transgenic promoter, (ii) new insights into the biology of the serosa, (iii) how expression in the mesodermal line G04609 compares to the expression of genes near the transposon insertion site, and (iv) some general features that make these lines valuable visualization tools. Our analyses also involved the definition of morphological landmarks as well as their timing and variability under live imaging conditions (Fig. 1H, Table 1). As several of the following conclusions depend on precise information on developmental staging and age, these quantifications are also a key way in which this study lays the foundation for future live imaging analyses across *Tribolium* embryogenesis.

### Shared expression domains across lines, and contribution of the 3xP3 promoter

The artificial 3xP3 promoter within the transposon serves as a transgenic marker across all life history stages and is generally referred to as an eye marker, as the original description of expression encompasses photoreceptors and the optic nerve [29]. However, additional postembryonic expression domains in the nervous system were already known for *Tribolium* and several fly species [22], as was a number of nervous system domains, the hindgut, and anal plates in the pre-hatching *Drosophila* embryo (stage 17; [30]). Consistent with these observations, during *Tribolium* embryogenesis we also detect EGFP in the eyes, optic nerve, brain, and ventral nerve cord in all three lines (Figs. 2D,E, 5B1). We did not observe hindgut expression nor clear anal expression, although the latter domain may have low level expression that we have not distinguished from the posteriormost portion of the nerve cord or of the G04609 line-specific, segmentally iterated expression (Figs. 2D, 5A–B). However, as described above, brain and ventral nerve cord expression arises earlier and is stronger in the KT650 line, suggesting that this is a genuine component of the line’s expression profile, independent of the later 3xP3-driven expression common to all three lines.

Even when 3xP3-driven expression domains are discounted, all three lines examined in this study show expression in yolk globules (Figs. 1C1,E1, 4A1–A2), the serosa (Figs. 1, 2, 4A3–B3), and diverse leg domains (Figs. 2D–E, 4, 5).

The yolk expression is a weak and predominantly early feature whose amorphous character is not readily attributable to either the 3xP3 or endogenous enhancers’ other expression domains. Yolk energids – nuclei and their surrounding cytoplasmic islands – are known in *Tribolium* [14], [17] and visible with the nGFP line, but are much more numerous and smaller than the EGFP-labeled globules. As nothing similar to the yolk globules is visible in the nGFP line, we exclude the possibility of an autofluorescence or visualization artifact. On the other hand, the shape of the yolk globules observed here does not correspond to the “yolk spheres” described previously [3], [31]. Also, as globule size is variable in KT650 (Fig. 1E1), it seems that the EGFP is not labeling a consistent, single anatomical structure. Given the speculation on the roles of yolk structures in early development in these cited studies, here we merely document this minor, peculiar aspect of the enhancer trap lines’ expression patterns for future evaluation, as by optical sectioning of fixed early preparations to visualize EGFP in conjunction with yolk structural components [3], [31].

We have presented G12424 and KT650 as serosal lines but also note that the predominantly mesodermal marking line G04609 shows persistent serosal expression. Indeed, we think that the serosal expression documented for G04609 does reflect actual enhancer trap driven expression. Other lines that we have investigated do not show serosal expression (unpublished data), indicating that it is not a universal feature. Also, the case of G04609 may not be so unusual in its combined serosal and mesodermal expression domains, as *Tc-mid* also has extraembryonic expression (Fig. 7B,E). Although *Dm-mid* is not expressed in the amnioserosa, dual heart and amnioserosal expression is known for the upstream gene *pannier* as well as for several fly enhancer trap lines [32].

Lastly, the embryonic legs are an anatomically complex structure with EGFP expression in all three lines examined here. As the exact location and shape of the expression domains differs across the lines (Figs. 2D–E, 4B, 5A–B), we interpret these as bona fide and unique domains.

### Insights into serosal development: regionalization and expression time courses

As explained above, the complex biology of the typical insect serosa [18], [33]–[38] does not readily lend itself to predictions of candidate marker genes based solely on orthologous gene expression in the *Drosophila* amnioserosa. Furthermore, in many insects such as *Tribolium*, the serosa’s own cuticle has made analyses based on whole mount staining untenable across a large interval of embryogenesis. Here, we present serosal enhancer trap lines that together clearly label the tissue from its definitive formation through its demise, making it readily visible and thus amenable to detailed examination. For example, all three lines reveal new structural features of the tissue, while the two serosa-specific lines’ expression profiles likely reflect distinct aspects of serosal biology.

One intriguing aspect of the onset of serosal EGFP in all three lines is the asymmetry of early expression, with stronger anterior-dorsal, anterior-central, or anterior-ventral and dorsal domains (Figs. 1C2,E2, A3–A4, respectively). Put another way, the ventral-posterior region is the last to develop serosal EGFP expression. This may simply correlate with small temporal differences in maturation across the tissue, as the portion involved in the closure of the serosal window over the embryo and amnion (ventral-central region: Fig. 1A3; [3], [14]) is the last region to comprise the complete serosal sheet (following separation from the amnion and concomitant intra-tissue serosal fusion). On the other hand, serosal EGFP expression is already uniform in the early-expressing G12424 line at the same time as it is initially asymmetric in the late-expressing KT650 (compare Fig. 1C4 and 1E2). Furthermore, although serosal expression in the ‘mesodermal’ line G04609 remains relatively weak, in contrast to the other lines it also retains the anterior expression bias throughout development, even during serosal folding and compaction (Fig. 4B1–B3). As the known serosal markers in *Tribolium* are mostly expressed throughout the tissue [15], [39], while the asymmetries seen here approximately correlate with the sites of serosal window closure and later serosal rupture, such regionalized expression will be key in future dissection of the serosa’s morphogenetic repertoire.

A second aspect of serosal regionalization pertains to the morphological change in the intracellular distribution of EGFP that begins during early germband retraction (dynamic streaks of EGFP: Fig. 1C4′). As this seems restricted to the portion of the serosa over the yolk rather than the amnion (Fig. 2C1′), we present the first indication that the behavior of the superficially uniform serosa is influenced by its diverse substrates.

In addition to revealing this new morphological feature, the EGFP distribution throughout the squamous serosal cells shows the nucleus and gives a good approximation of the cell’s area and shape (Fig. 2A–C). In understanding the cellular basis for tissue reorganization, this provides a needed level of detail [40]. Due to the EGFP protein perdurance, the tissue also remains fluorescently labeled even through the stages of final degeneration via apoptosis (Fig. 1G3, [16]). It is interesting to observe that in *Tribolium* the serosa begins to fragment and leave behind cytoplasmic trails (Fig. 6B,C3) even at relatively early stages of contraction, in contrast to other insect species in which the serosa contracts with a different relative topography and maintains tissue integrity for longer [41]–[43].

Despite the common utility of both serosal lines for mature tissue labeling, the overall time courses of EGFP expression levels (Fig. 1H) reveal distinct regulatory environments of the two insertion sites. The strong, rapid increase in EGFP levels in the early-expressing G12424 line occurs just after the serosal window has closed, heralding the maturation of the serosa as a complete extraembryonic cover. That G12424 expression levels build for some time (over 17 hours, or 24% of embryogenesis), likely represents the turning point between accumulation of EGFP protein from new enhancer-driven transcription and low rates of protein degradation. Despite the gradual wane in EGFP signal, it remains readily detectable through final serosal degradation, making this line both an indicator of initial tissue maturation and a persistent tissue marker. In contrast, the late-expressing KT650 line shows a gradual increase in EGFP signal throughout the time the serosa remains a complete extraembryonic cover (between serosal window closure and rupture stages). This would be consistent with low, steady-state levels of transcription that result in gradual EGFP protein accumulation and that reflect ongoing physiological processes, perhaps such as endoreplication [34], vesicular trafficking [35], [36], water regulation [33], or indeed cuticle production ([18], [36]; Table 3, gene #5). Given that several of the genes near to both lines’ insertion sites are of unknown function or lack homologues in *Drosophila* (Tables 2,3), the eventual elucidation of the enhancer trap molecular environment has the potential to reveal genuinely new aspects of serosal tissue regulation.

### Evaluation of G04609 expression in comparison with the neighboring genes *Tc-mid* and *Tc-H15*

Our identification of the EGFP expression domains in the G04609 line corroborates and extends a previous characterization of this line. Based on *in situ* hybridization detection of EGFP transcript in flat mounted, retracted germband embryos, expression was also observed in the legs, lateral segmental domains, and the heart [44]. Our live imaging analyses across the second half of embryogenesis demonstrate that these domains continuously express EGFP (Fig. 4). Interestingly, while the initially broad distal expression in the legs refines to a multidomain pattern (Fig. 5), expression in the cardioblast cell row of the heart is extremely stable, including at postembryonic stages (Figs. 4, 6). The stable heart expression is surprising in that it is irregular: a given cardioblast does or does not express EGFP (Fig. 6D1–D5), but at no stage did we observe either that all cardioblasts express EGFP or that the on-off pattern represents any kind of hemisegmentally repeated pattern. This differs from *midline* and *H15* expression in both *Tribolium* and *Drosophila*. In our analyses, all cardioblasts expressed *Tc-mid*, while *Tc-H15* expression was continuous but consistently with a posterior-high anterior-low gradient in late development (Fig. 7D1,I1). In *Drosophila*, initial *Dm-mid* expression occurs in two cells per hemisegment, after which both genes are expressed in all cardioblasts [45]–[47].

As stated in the Results section, we conclude that the G04609 expression pattern is more similar to that of *Tc-mid* than *Tc-H15.* Although it is beyond the scope of the current study to make a full examination of *Tc-mid* and *Tc-H15*, we note that whereas in *Drosophila* both genes are expressed in segmental stripes in early development, we only observe this expression pattern for *Tc-H15*, and not for *Tc-mid* or for G04609 EGFP (Fig. 7B,G). Secondly, even when cardioblast G04609 EGFP is weak at the onset (Fig. 4B1), at no time do we observe the posterior-to-anterior gradient of expression seen for *Tc-H15* (Fig. 7I1). Thirdly, although early G04609 EGFP expression in the legs encompasses a relatively broad domain (Fig. 5A, [44]), this later refines to a subset of distinct structures (Fig. 5B1,B3). The later expression pattern is more akin to that of *Tc-mid* (Fig. 7D3) than to *Tc-H15*, as the latter remains broadly expressed in the legs (Fig. 7I3), similar to what was previously observed in younger embryos [28].

Lastly, G04609 expression occurs in the serosa (Fig. 4A3–B3), and *Tc-mid* also exhibits extraembryonic expression whereas *Tc-H15* never does so above background levels (Fig. 7B,E,G,I1). We are somewhat cautious in equating the early extraembryonic expression of G04609 and *Tc-mid*, however. Although the asymmetric expression patterns are temptingly similar for the dorsal-anterior region (compare Figs. 4A3 and 7B), the *Tc-mid* early expression is highly dynamic in the pre- to uniform blastoderm stages before switching off, making the embryo shown somewhat of an outlier (Fig. 7B). Also, even if the early *Tc-mid* expression can be spatially reconciled with an unambiguously serosal domain, the transcript is no longer detectable four to seven hours before G04609 EGFP is first seen in this domain, commencing after the serosal window has already closed (Fig. 4A3).

Although the artificial 3xP3 core promoter is recognized as being very efficient in driving expression in *Tribolium*, a delay of over two hours has been documented between induction of transcription and clearly discernible fluorescence in embryos, even when using the TurboGFP variant that matures more rapidly than EGFP [2], [48]. To more directly compare the G04609 enhancer trap expression with that of *Tc-mid* transcripts in early development, we performed *in situ* hybridization for *egfp*. However, two separate experiments failed to recapitulate the *Tc-mid* expression in early embryos. Specific stain was weakly detected in broad segmental blocks of the young germband, with patchy anterior-dorsal serosal expression first detectable when the germband had already reached 100% egg length (roughly, just after closure of the serosal window). Hence *egfp* transcript detection is spatially and temporally very similar to EGFP fluorescent signal detection for the serosa, while for the presumptive segmental muscle blocks the transcript signal seems to arise some time before clear chromaphore detection.

As for the late *Tc-mid* extraembryonic expression, it appears to encompass the amnion as well as serosa and to be relatively strong in comparison with mesodermal expression domains, whereas G04609 is not expressed in the amnion and the late serosal expression is relatively weak (Fig. 4B3). As noted above, a final difference is that *Tc-mid* is expressed in all cardioblasts whereas G04609 is not.

Despite these caveats, *Tc-mid* is the nearest gene to the G04609 insertion site, and the two share a number of expression features. Given the modular nature of enhancers and the potential for subtle positional effects, we suggest that G04609 is trapping a subset of the full *cis* regulatory information driving *Tc-mid* expression, and that some of this subset may be under the control of an enhancer shared between *Tc-mid* and *H-15*. However, as we are examining enhancer traps rather than exon or other more direct gene traps, identifying endogenous genes with similar expression patterns does not fully clarify the identity, location, or number of enhancer(s) acting on both the transposon and on the endogenous gene over potentially large genomic distances. Our results come at the beginning of such detailed investigations.

### Key properties of the enhancer trap lines and research outlook

To conclude, we will address how some of the more implicit features of our results demonstrate the strength of the enhancer trap lines G12424, KT650, and G04609 in particular and as representative lines for the entire enhancer trap collection [5].

The lines’ usefulness for visualization stems from the strength of the EGFP signal, which has three beneficial consequences. Firstly, that the lines are readily detectible for live imaging makes them powerful tools for watching the dynamic progression of development. To the best of our knowledge, the handful of studies making use of enhancer traps from this collection have thus far only visualized expression in fixed tissues (*e.g.*, [44]). Secondly, even in fixed specimens the EGFP signal can be detected without any immunochemical amplification. Thirdly, both of these features hold true even when different lines are crossed to produce heterozygote offspring, enabling the simultaneous labeling of multiple tissue domains and thus also demarcating intervening structures (Figs. 2A, 6A). We found that either serosal line worked equally well when crossed with G04609, regardless of whether the mother or father provided a particular enhancer trap.

As stated at the outset, our primary aim in selecting these three lines was to find useful embryonic dorsal and extraembryonic markers for examination of serosal rupture through dorsal closure stages to further our previous work [16]. In this regard, the labeled heart in the G04609 line provides a discrete structure that relatively closely approximates the position of the dorsal ectoderm, as it lies directly below the ectoderm and only about 3–4 cell rows back from the ectodermal-amniotic boundary (Fig. 6A–C,G; [16]). Furthermore, as dorsal closure proceeds the cardioblast row increasingly shows small bends that are comparable to the amnion-epidermis inter-tissue border shape (Fig. 6D1–D5; [16]). Thus a serosa × heart EGFP cross also serves to delimit the amniotic region during dorsal closure (Figs. 2A, 6A), effectively labeling both extraembryonic tissues and their boundary with the embryo with a single fluorescent label. In this regard, it is again advantageous that the EGFP is not localized within the cells (it is not part of a fusion protein, and unlike the nGFP line there is no localization sequence tag), so that the fluorescent signal fully illuminates a given tissue (*e.g.*, Fig. 2B). In sum, all three lines may be useful in future morphogenetic analyses of RNA interference (RNAi) knockdown phenotypes with dorsal closure and related defects (*e.g.*, [49]).

In addition to the indirect visualization of unlabeled tissues, composite expression domains – whether from heterozygotes or a single line – also provide a number of anatomical landmarks for phenotypic analyses. For example, the G04609 line provides discrete coordinate information along both the anterior-posterior (segmental muscles) and dorsal-ventral (legs, segmental muscles, heart) axes of the embryo, and these regions continue to be marked postembryonically (Fig. 4C3).

Finally, the ongoing quest to make use of the information on the genomic insertion sites in these lines will greatly expand our understanding of *Tribolium* development. The initial identification of the endogenous genes with expression patterns corresponding to the enhancer trap EGFP, such as we have done here for G04609 in relation to *Tc-mid*, provides an unbiased means of screening for genes with desired expression patterns. Furthermore, the known integration sites can be used for targeted knock-in integration of new transgenic constructs for both localized fluorescent labeling and for mis-expression applications. When the actual enhancers that these lines have trapped are identified, this information will provide still greater spatial and temporal experimental control via additional drivers of the Gal4/UAS expression system [2]. With only approximately 5% of the complete set of 505 GEKU enhancer trap lines [5] characterized thus far, we look forward to the many biological insights that will ensue as this *Tribolium* resource is more fully tapped.

## Methods

### *Tribolium castaneum* beetle stocks

The GEKU enhancer trap lines have the pBac{3xP3-EGFPaf} transposon cassette inserted into in the *pearl* white-eyed mutant background [5], [22]. The lines G12424, KT650, and G04609 were chosen by screening the “GEKU base” website (http://www.geku-base.uni-goettingen.de; site last accessed 10 March 2014). For developmental staging calibration, live imaging was performed simultaneously with the transgenic line that ubiquitously expresses GFP with a nuclear localization sequence (nGFP line: [4]). For *in situ* hybridization, we used the wild type San Bernardino (SB) strain [50].

### Mapping and analysis of transposon insertion sites

The site of transposon insertion was determined by inverse PCR as in the original screening paper [5]. Genomic DNA (gDNA) was digested with HinP1 I, purified, and then circularized with T4 DNA ligase (both enzymes from New England Biolabs). Nested PCR was performed with transposon-specific primers targeting the 3′ end of the insertion. We obtained inverse PCR fragments containing 899 bp, 444 bp, and 1027 bp of specific genomic sequence from the G12424, KT650, and G04609 lines, respectively. The same procedure applies after Sau3A I digestion when using primers for the 5′ end of the transposon, but this was less robust in our hands. The insertion site was then confirmed via PCR with specific primers for the flanking gDNA on either side of the transposon in combination with primers for the 5′ and 3′ ends of the transposon. For these analyses both the Tcas_3.0 and Assembly 4.0 genome versions were used, as accessed from the genome browser of the Stanke group, University of Greifswald (http://bioinf.uni-greifswald.de/gb2/gbrowse/tcas4/).

To provide an initial evaluation of some of the genes near to the transposon insertion site, information was obtained on the *Drosophila melanogaster* orthologue or nearest homologue from FlyBase (http://flybase.org/, release FB2014_03 and earlier) and the Berkeley Drosophila Genome Project (BDGP, http://insitu.fruitfly.org/, [51]–[53]). Orthologues and homologues were determined from the BLAST functionality within the *Tribolium* genome browser.

### Live imaging

Embryos were dechorionated and mounted in hanging drops of halocarbon oil, as described previously [16]. Time-lapse recordings were acquired on an Applied Precision DeltaVision RT widefield microscope and on a Zeiss AxioImager.Z2 with an Apotome.2 module for structured illumination, at 30°C or 23.5±1°C, respectively. To ensure an accurate representation of embryogenesis, movie data were only used from embryos that successfully completed all developmental stages recorded in a given movie and did not show any physiological deterioration. After filming, embryos were re-examined several days later to confirm subsequent survival and hatching (hatching on the slide was not a strict requirement for samples mounted at the blastoderm stage). At 30°C, total embryogenesis is approximately 72 hours.

### Quantitative analyses of time-lapse movie data

Eggs were collected over a 4-hour interval; age is given in hours after egg lay (hAEL) for the lower end of the range (“minimum age”). For these analyses, images were acquired every ten minutes with the DeltaVision microscope at 30°C. The motorized stage enabled the simultaneous recording of all three lines and the nuclear-GFP line, which was used for staging calibration. Film 1 recorded younger eggs over a 20-hour interval (4.8–24.8 hAEL). Film 2 overlapped with Film 1 by 6.9 hours in recording older eggs for 46 hours (17.9–64.2 hAEL). Together, Films 1 and 2 spanned embryogenesis from the uniform blastoderm stage (4.8 hAEL, 7% development) until after dorsal closure (64.2 hAEL, 89%). Two separate films were recorded due to the logistics of using a shared imaging facility, and to ensure a sufficient sample size for the older stages (as embryos mounted at the blastoderm stage are somewhat fragile, not all of these are able to hatch after three days of constant filming with the conditions used here). For each line, sample sizes ranged from 7 to 11 embryos per film (specified in Figs. 1H, 4). Where data were available, embryos from all four transgenic lines (nGFP, G12424, KT650, G04609) were used for calculations of stage age (Table 1). For quantification of the EGFP expression time course, time-lapse images were analyzed in ImageJ software (NIH), where the integrated density was measured on maximum intensity projection images for polygonal regions of interest that fully encompassed a given egg. The “relative GFP signal” represents the integrated density divided by the area of the region of interest, where plotted values show the mean±1 standard deviation. In plotting the results from Films 1 and 2 on the same graph, the slight reduction in relative EGFP signal in Film 2 reflects a small reduction in exposure time during acquisition compared to Film 1, an optimization for preventing signal saturation. For tracking yolk globules (Fig. 4A2), the ImageJ plugin MTrackJ was used [54].

### Fixed embryo visualization and *in situ* hybridization

To visualize endogenous EGFP, embryos were fixed, hand dissected, post-fixed, and then mounted in Vectashield mountant with DAPI (Vector Laboratories) as previously described [16]. Images were acquired with a Zeiss LSM 700 laser scanning confocal.

For *in situ* hybridization, embryos were also prepared in this manner for older stages. Younger stages were similarly dechorionated and fixed (for 45–60 minutes), and then devitellinized by methanol shock. *In situ* hybridization was performed as previously described [10], with detection with NBT-BCIP precipitate (Roche). Probes were generated from cDNA templates with the primers specified in Table 4, for either sense or antisense transcription. In all cases the sense strand probe was used as a negative control and each probe was tested in at least three separate experiments. For *egfp*, antisense detection in the wild type (SB), non-transgenic background served as an additional negative control. Probes for previously characterized genes were used as positive controls. All probes were synthesized with the DIG RNA Labeling Mix and T7 RNA polymerase (Roche). Stained embryos were also mounted in Vectashield with DAPI, and images were acquired with a Zeiss Axioplan2 or a Zeiss AxioImager.Z2 with Apotome.2 microscope. Helicon Focus software (v. 4.2.9 for Mac, HeliconSoft Ltd.) was used to generate projected images of selected micrographs (Figs. 2D–E, 7D–J).

## Supporting Information

Movie S1 **Movie of late serosal morphogenesis, visualized with the KT650 serosal line (see also** **Figure 2C** **1–C3).** The time-lapse movie spans the stages from pre-rupture through compaction to form the dorsal organ and through to the beginning of serosal degeneration. The embryo is in dorsal-lateral aspect with anterior left and dorsal up. The image is a maximum intensity projection for EFGP signal (green) overlaid on a brightfield image (grey channel: embryonic tissue is light grey; the opaque yolk is black). The time stamp shows time in minutes relative to serosal rupture ( = 0 min), from filming at 23.5±1°C. Scale bar is 100 µm.
(AVI)

Movie S2 **Movie of mesodermal EGFP expression development in the line G04609 (see also** **Figure 4B** **1–B4).** The embryo is shown from mid germband extension through the completion of dorsal closure and onset of muscle twitches in this 46-hour time-lapse movie. The embryo is in lateral aspect with anterior left and dorsal up. The image is a maximum intensity projection for EFGP signal (white). The time stamp shows embryo age in hours after egg lay (hAEL: 17.9 to 64.2), from filming at 30°C. Scale bar is 100 µm.
(AVI)

## References

[1] Schinko JB, Hillebrand K, Bucher G (2012) Heat shock-mediated misexpression of genes in the beetle *Tribolium castaneum* . Dev Genes Evol 222: 287–298. 22890852 10.1007/s00427-012-0412-x

[2] Schinko JB, Weber M, Viktorinova I, Kiupakis A, Averof M, et al (2010) Functionality of the GAL4/UAS system in *Tribolium* requires the use of endogenous core promoters. BMC Dev Biol 10: 53. 20482875 10.1186/1471-213X-10-53 PMC2882914

[3] Benton MA, Akam M, Pavlopoulos A (2013) Cell and tissue dynamics during *Tribolium castaneum* embryogenesis revealed by versatile fluorescence labeling approaches. Development 140: 3210–3220. 23861059 10.1242/dev.096271 PMC3930475

[4] Sarrazin AF, Peel AD, Averof M (2012) A segmentation clock with two-segment periodicity in insects. Science 336: 338–341. 22403177 10.1126/science.1218256

[5] Trauner J, Schinko J, Lorenzen MD, Shippy TD, Wimmer EA, et al (2009) Large-scale insertional mutagenesis of a coleopteran stored grain pest, the red flour beetle *Tribolium castaneum*, identifies embryonic lethal mutations and enhancer traps. BMC Biol 7: 73. 19891766 10.1186/1741-7007-7-73 PMC2779179

[6] *Tribolium* Genome Sequencing Consortium (2008) The genome of the model beetle and pest *Tribolium castaneum* . Nature 452: 949–955. 18362917 10.1038/nature06784

[7] Beermann A, Prühs R, Lutz R, Schröder R (2011) A context-dependent combination of Wnt receptors controls axos elongation and leg development in a short germ insect. Development 138: 2793–2805. 21652652 10.1242/dev.063644 PMC3188592

[8] Kittelmann S, Ulrich J, Posnien N, Bucher G (2013) Changes in anterior head patterning underlie evolution of long germ embryogenesis. Dev Biol 374: 174–184. 23201022 10.1016/j.ydbio.2012.11.026

[9] El-Sherif E, Averof M, Brown SJ (2012) A segmentation clock operating in blastoderm and germband stages of *Tribolium* development. Development 139: 4341–4346. 23095886 10.1242/dev.085126 PMC3509729

[10] Nunes da Fonseca R, von Levetzow C, Kalscheuer P, Basal A, van der Zee M, et al (2008) Self-regulatory circuits in dorsoventral axis formation of the short-germ beetle *Tribolium castaneum* . Dev Cell 14: 605–615. 18410735 10.1016/j.devcel.2008.02.011

[11] Grossmann D, Prpic N-M (2012) Egfr signaling regulates distal as well as medial fate in the embryonic leg. Dev Biol 370: 264–272. 22921411 10.1016/j.ydbio.2012.08.005

[12] Schmitt-Engel C, Cerny AC, Schoppmeier M (2012) A dual role for *nanos* and *pumilio* in anterior and posterior blastodermal patterning of the short-germ beetle *Tribolium castaneum* . Dev Biol 364: 224–235. 22326441 10.1016/j.ydbio.2012.01.024

[13] Panfilio KA (2008) Extraembryonic development in insects and the acrobatics of blastokinesis. Dev Biol 313: 471–491. 18082679 10.1016/j.ydbio.2007.11.004

[14] Handel K, Grünfelder CG, Roth S, Sander K (2000) *Tribolium* embryogenesis: a SEM study of cell shapes and movements from blastoderm to serosal closure. Dev Genes Evol 210: 167–179. 11180819 10.1007/s004270050301

[15] van der Zee M, Berns N, Roth S (2005) Distinct functions of the *Tribolium zerknüllt* genes in serosa specification and dorsal closure. Curr Biol 15: 624–636. 15823534 10.1016/j.cub.2005.02.057

[16] Panfilio KA, Oberhofer G, Roth S (2013) High plasticity in epithelial morphogenesis during insect dorsal closure. Biol Open 2: 1108–1118. 24244847 10.1242/bio.20136072 PMC3828757

[17] Stanley MSM, Grundmann AW (1970) The embryonic development of *Tribolium confusum* . Ann Entomol Soc Amer 63: 1248–1256.

[18] Jacobs CGC, Rezende GL, Lamers GEM, van der Zee M (2013) The extraembryonic serosa protects the insect egg against desiccation. Proc R Soc B 280: 20131082. 10.1098/rspb.2013.1082 PMC3712428 23782888

[19] Berghammer A, Bucher G, Maderspracher F, Klingler M (1999) A system to efficiently maintain embryonic lethal mutations in the flour beetle *Tribolium castaneum* . Dev Genes Evol 209: 382–389. 10370121 10.1007/s004270050268

[20] Horn C, Wimmer EA (2000) A versatile vector set for animal transgenesis. Devel Genes Evol 210: 630–637. 11151300 10.1007/s004270000110

[21] Handel K, Basal A, Fan X, Roth S (2005) *Tribolium castaneum twist*: gastrulation and mesoderm formation in a short-germ beetle. Dev Genes Evol 215: 13–31. 15645317 10.1007/s00427-004-0446-9

[22] Lorenzen MD, Berghammer AJ, Brown SJ, Denell RE, Klingler M, et al (2003) *piggyBac*-mediated germline transformation in the beetle *Tribolium castaneum* . Insect Mol Biol 12: 433–440. 12974948 10.1046/j.1365-2583.2003.00427.x

[23] Broadie K, Bellen HJ, DiAntonio A, Littleton JT, Schwarz TL (1994) Absence of synaptotagmin disrupts excitation-secretion coupling during synaptic transmission. Proc Natl Acad Sci USA 91: 10727–10731. 7938019 10.1073/pnas.91.22.10727 PMC45095

[24] Yoshihara M, Montana ES (2004) The Synaptotagmins: Calcium sensors for vesicular trafficking. Neuroscientist 10: 566–574. 15534041 10.1177/1073858404268770

[25] Arakane Y, Hogenkamp DG, Zhu YC, Kramer KJ, Specht CA, et al (2004) Characterization of two chitin synthase genes of the red flour beetle, *Tribolium castaneum*, and alternate exon usage in one of the genes during development. Insect Biochem Mol Biol 34: 291–304. 14871625 10.1016/j.ibmb.2003.11.004

[26] Arakane Y, Muthukrishnan S, Kramer KJ, Specht CA, Tomoyasu Y, et al (2005) The *Tribolium* chitin synthase genes *TcCHS1* and *TcCHS2* are specialized for synthesis of epidermal cuticle and midgut peritrophic matrix. Insect Mol Biol 14: 453–463. 16164601 10.1111/j.1365-2583.2005.00576.x

[27] Olson EN (2006) Gene regulatory networks in the evolution and development of the heart. Science 313: 1922–1927. 17008524 10.1126/science.1132292 PMC4459601

[28] Janssen R, Damen WGM (2008) Diverged and conserved aspects of heart formation in a spider. Evol Dev 10: 155–165. 18315809 10.1111/j.1525-142X.2008.00223.x

[29] Berghammer AJ, Klingler M, Wimmer EA (1999) A universal marker for transgenic insects. Nature 402: 370–371. 10586872 10.1038/46463

[30] Horn C, Jaunich B, Wimmer EA (2000) Highly sensitive, fluorescent transformation marker for *Drosophila* transgenesis. Dev Genes Evol 210: 623–629. 11151299 10.1007/s004270000111

[31] Peel AD, Averof M (2010) Early asymmetries in maternal transcript distribution associated with a cortical microtubule network and a polar body in the beetle *Tribolium castaneum* . Dev Dyn 239: 2875–2887. 20857499 10.1002/dvdy.22423

[32] Jin H, Stojnic R, Adryan B, Ozdemir A, Stathopoulos A, et al (2013) Genome-wide screens for in vivo Tinman binding sites identify cardiac enhancers with diverse functional architectures. PLoS Genet 9: e1003195. 23326246 10.1371/journal.pgen.1003195 PMC3542182

[33] Mori H (1972) Water absorption by the columnar serosa in the eggs of the waterstrider, *Gerris paludum insularis* . J Insect Physiol 18: 675–681.

[34] Truckenbrodt W (1973) Über die Entstehung der Serosa im besamten und im unbesamten Ei von *Odontotermes badius* Hav. (Insecta, Isoptera). Z Morph Tiere 76: 193–208.

[35] Dorn A (1976) Ultrastructure of embryonic envelopes and integument of *Oncopeltus fasciatus* Dallas (Insecta, Heteroptera) I. Chorion, amnion, serosa, integument. Zoomorphologie 85: 111–131.

[36] Lamer A, Dorn A (2001) The serosa of *Manduca sexta* (Insecta, Lepidoptera): ontogeny, secretory activity, structural changes, and functional considerations. Tissue Cell 33: 580–595. 11827102 10.1054/tice.2001.0213

[37] Chen G, Handel K, Roth S (2000) The maternal NF-kappaB/Dorsal gradient of *Tribolium castaneum*: dynamics of early dorsoventral patterning in a short-germ beetle. Development 127: 5145–5156. 11060240 10.1242/dev.127.23.5145

[38] Jacobs CGC, van der Zee M (2013) Immune competence in insect eggs depends on the extraembryonic serosa. Dev Comp Immunol 41: 263–269. 23732406 10.1016/j.dci.2013.05.017

[39] Sharma R, Beermann A, Schröder R (2013) The dynamic expression of extraembryonic marker genes in the beetle *Tribolium castaneum* reveals the complexity of serosa and amnion formation in a short germ insect. Gene Expr Patt 13: 362–371. 10.1016/j.gep.2013.07.002 23856408

[40] Blanchard GB, Kabla AJ, Schultz NL, Butler LC, Sanson B, et al (2009) Tissue tectonics: morphogenetic strain rates, cell shape change and intercalation Nat Methods. 6: 458–464. 10.1038/nmeth.1327 PMC4894466 19412170

[41] Panfilio KA (2009) Late extraembryonic development and its *zen-RNAi*-induced failure in the milkweed bug *Oncopeltus fasciatus* . Dev Biol 333: 297–311. 19580800 10.1016/j.ydbio.2009.06.036

[42] Panfilio KA, Roth S (2010) Epithelial reorganization events during late extraembryonic development in a hemimetabolous insect. Dev Biol 340: 100–115. 20045678 10.1016/j.ydbio.2009.12.034

[43] Enslee EC, Riddiford LM (1981) Blastokinesis in embryos of the bug, *Pyrrhocoris apterus*. A light and electron microscopic study 1. Normal blastokinesis. J Embryol Exp Morph 61: 35–49. 7264550

[44] Grossmann D, Scholten J, Prpic N-M (2009) Separable functions of *wingless* in distal and ventral patterning of the *Tribolium* leg. Dev Genes Evol 219: 469–479. 20024581 10.1007/s00427-009-0310-z PMC2811246

[45] Miskolczi-McCallum CM, Scavetta RJ, Svendsen PC, Soanes KH, Brook WJ (2005) The *Drosophila melanogaster* T-box genes *midline* and *H15* are conserved regulators of heart development. Dev Biol 278: 459–472. 15680363 10.1016/j.ydbio.2004.11.026

[46] Qian L, Liu J, Bodmer R (2005) *Neuromancer* Tbx20-related genes (*H15/midline*) promote cell fate specification and morphogenesis of the *Drosophila* heart. Dev Biol 279: 509–524. 15733676 10.1016/j.ydbio.2005.01.013

[47] Reim I, Mohler JP, Frasch M (2005) *Tbx20*-related genes, *mid* and *H15*, are required for tinman expression, proper patterning, and normal differentiation of cardioblasts in *Drosophila* . Mech Dev 122: 1056–1069. 15922573 10.1016/j.mod.2005.04.006

[48] Evdokimov AG, Pokross ME, Egorov NS, Zaraisky AG, Yampolsky IV, et al (2006) Structural basis for the fast maturation of Arthropoda green fluorescent protein. EMBO Rep 7: 1006–1012. 16936637 10.1038/sj.embor.7400787 PMC1618374

[49] Sharma R, Beermann A, Schröder R (2013) FGF signalling controls anterior extraembryonic and embryonic fate in the beetle *Tribolium* . Dev Biol 381: 121–133. 23769707 10.1016/j.ydbio.2013.05.031

[50] Brown SJ, Shippy TD, Miller S, Bolognesi R, Beeman RW, et al (2009) The red flour beetle, *Tribolium castaneum* (Coleoptera): A model for studies of development and pest biology. Cold Spring Harb Protoc 2009: pdb.emo126. 20147228 10.1101/pdb.emo126

[51] Tomancak P, Beaton A, Weiszmann R, Kwan E, Shu S, et al (2002) Systematic determination of patterns of gene expression during *Drosophila* embryogenesis. Genome Biol 3: RESEARCH0088. 12537577 10.1186/gb-2002-3-12-research0088 PMC151190

[52] Tomancak P, Berman BP, Beaton A, Weiszmann R, Kwan1 E, et al (2007) Global analysis of patterns of gene expression during *Drosophila* embryogenesis. Genome Biol 8: R145. 17645804 10.1186/gb-2007-8-7-r145 PMC2323238

[53] Hammonds AS, Bristow CA, Fisher WW, Weiszmann R, Wu S, et al (2013) Spatial expression of transcription factors in *Drosophila* embryonic organ development. Genome Biol 14: R140. 24359758 10.1186/gb-2013-14-12-r140 PMC4053779

[54] Meijering E, Dzyubachyk O, Smal I (2012) Chapter 9: Methods for cell and particle tracking. In: Conn PM, editor. Imaging and Spectroscopic Analysis of Living Cells – Optical and Spectroscopic Techniques Elsevier. 183–200. 10.1016/B978-0-12-391857-4.00009-4 22264535

[55] Akay T, Bässler U, Gerharz P, Büschges A (2001) The role of sensory signals from the insect coxa-trochanteral joint in controlling motor activity of the femur-tibia joint. J Neurophysiol 85: 594–604. 11160496 10.1152/jn.2001.85.2.594

[56] Zill SN (1985) Plasticity and proprioception in insects. I. Responses and cellular properties of individual receptors of the locust metathoracic femoral chordotonal organ. J Exp Biol 116: 435–461. 4056657 10.1242/jeb.116.1.435

